# 3D-Printed Flat-Bone-Mimetic Bioceramic Scaffolds for Cranial Restoration

**DOI:** 10.34133/research.0255

**Published:** 2023-10-26

**Authors:** Yihang Zhang, Fupo He, Qiang Zhang, Haotian Lu, Shengtao Yan, Xuetao Shi

**Affiliations:** ^1^ School of Electromechanical Engineering, Guangdong University of Technology, Guangzhou 510006, P. R. China.; ^2^ School of Materials Science and Engineering, South China University of Technology, Guangzhou 510641, P. R. China.; ^3^ Peking Union Medical College Graduate School, Beijing 100730, P. R. China.; ^4^ Department of Emergency, China-Japan Friendship Hospital, Beijing 100029, P. R. China.

## Abstract

The limitations of autologous bone grafts necessitate the development of advanced biomimetic biomaterials for efficient cranial defect restoration. The cranial bones are typical flat bones with sandwich structures, consisting of a diploe in the middle region and 2 outer compact tables. In this study, we originally developed 2 types of flat-bone-mimetic β-tricalcium phosphate bioceramic scaffolds (Gyr-Comp and Gyr-Tub) by high-precision vat-photopolymerization-based 3-dimensional printing. Both scaffolds had 2 outer layers and an inner layer with gyroid pores mimicking the diploe structure. The outer layers of Gyr-Comp scaffolds simulated the low porosity of outer tables, while those of Gyr-Tub scaffolds mimicked the tubular pore structure in the tables of flat bones. The Gyr-Comp and Gyr-Tub scaffolds possessed higher compressive strength and noticeably promoted in vitro cell proliferation, osteogenic differentiation, and angiogenic activities compared with conventional scaffolds with cross-hatch structures. After implantation into rabbit cranial defects for 12 weeks, Gyr-Tub achieved the best repairing effects by accelerating the generation of bone tissues and blood vessels. This work provides an advanced strategy to prepare biomimetic biomaterials that fit the structural and functional needs of efficacious bone regeneration.

## Introduction

The cranial bone in the human body performs very important functions, such as protecting the brain and enabling the passage of the cranial nerves that are essential to physiological functioning [1,2]. Critical-sized cranial defects are commonly secondary to trauma, congenital disease, stroke, tumor, and aneurysms [1,3]. Cranial defects can cause encephalocele and osteosclerosis, and the resulting deformities disrupt the psychosocial well-being of patients [1,4,5]. Restoration of critical-sized cranial defects by cranioplasty is challenging for reconstructive surgeons, who prefer to use autologous bone grafts [1]. Autologous bone grafts have osteoconduction, osteoinduction, and revascularization capacities [6]. However, autologous bone grafting has a finite supply and requires additional surgeries concomitant with risks such as free flap loss, infection, deep venous thrombosis, and nerve injury [7]. These limitations necessitate the development of alternatives to autologous bone grafts for cranial defect restoration [8].

Biomaterials mimicking the composition and microstructure of natural bone are widely acknowledged to be ideal for bone defect regeneration [9–11]. Mimicking bone composition has a long history [12,13]. Calcium phosphates are the dominant component (~65 wt%) in natural bone [12]. As a representative calcium phosphate material, β-tricalcium phosphate (β-TCP) bioceramics are biodegradable, biocompatible, and osteoconductive, and they have received much research interest and many clinical applications [14]. In the past several decades, scientists have adjusted and optimized the pore structure and mechanical strength of biomaterials to promote bone formation and angiogenesis of bone defects at various sites, on the basis of simulating pore architecture features (3-dimensional [3D] interconnection, porosity, pore size, hierarchical structure, etc.) of natural bones [14–17]. These structure-mimicking biomaterials can be fabricated by conventional methods (gas forming, templating, freeze casing, etc.) and 3D printing techniques [18–20]. In recent years, an increasing number of studies have demonstrated that pore surface morphology plays a important role in bone formation and angiogenesis [21–25]. Unfortunately, the pore surface morphology of natural bone cannot be accurately reproduced and modified by traditional methods. Extrusion-type 3D printing is the most common method for fabricating porous biomaterials. However, the nozzle shape of the 3D printer limits the pore morphology, which is generally shown as a convex surface [19]. Vat photopolymerization (VPP)-type 3D printing is implemented by photopolymerization of ceramic/resin slurry layer by layer [26]. VPP printing allows for the fabrication of scaffolds with high precision and complicated pore architectures [27–30]. For example, Zhang et al. [28] used VPP-based 3D printing to fabricate hydroxyapatite bioceramic scaffolds with a triply periodic minimal surface pore topology. Feng et al. [29] prepared conch-mimicking bioceramic scaffolds with spiral structures capable of inducing directional migration of cells.

**Fig. 1.**
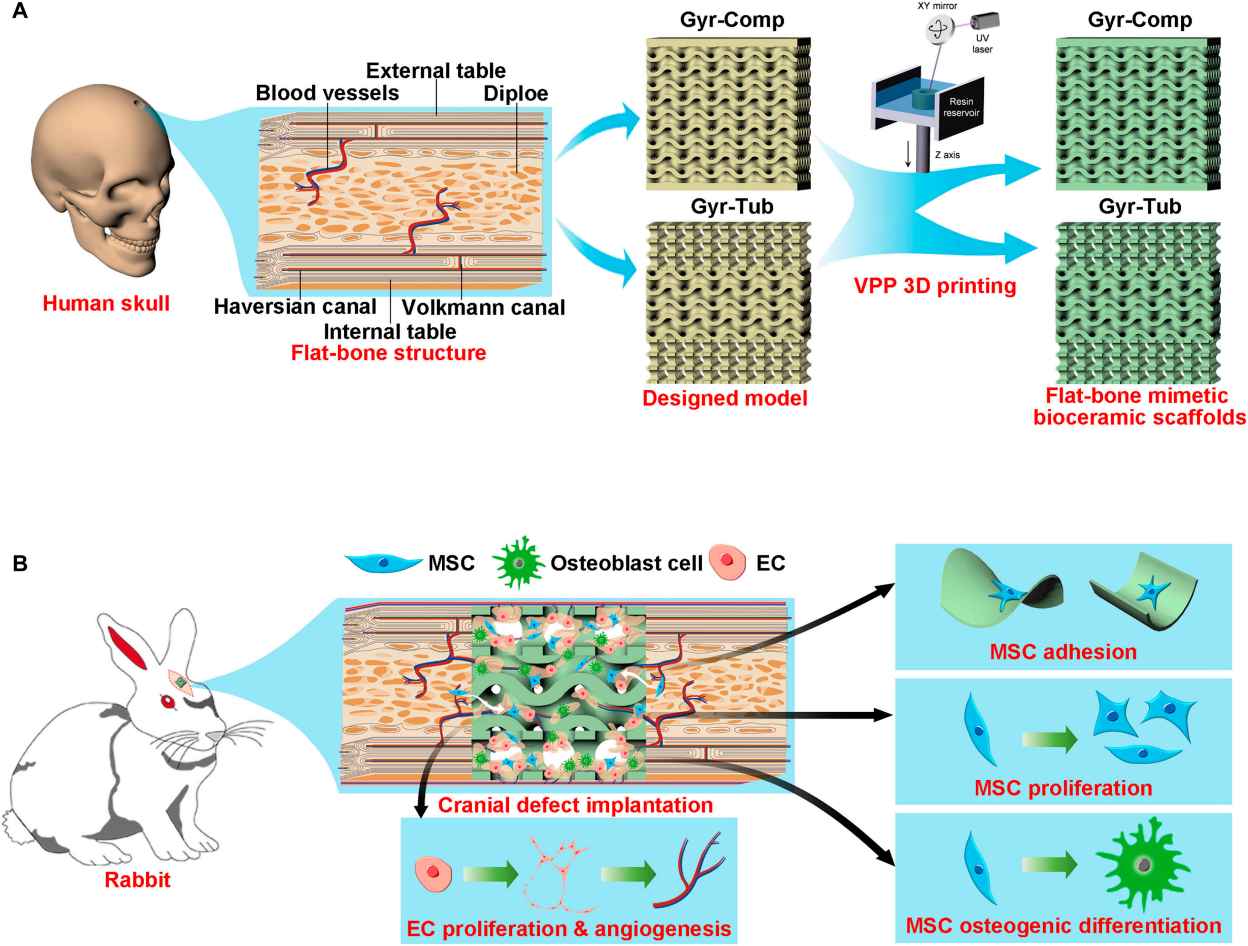
Schematic illustration of the design and bone regeneration potential of the flat-bone-mimetic bioceramic scaffolds. (A) The scaffolds are inspired by the sandwich structure of flat bone of craniums. (B) The scaffolds can provide a biomimetic structural environment to promote osteogenesis and angiogenesis. UV, ultraviolet. MSC, mesenchymal stromal cell; EC, endothelial cell.

The cranial bones are typical flat bones, which are generally thin and broad with a flattened or curved surface. A flat bone presents a sandwich structure [2]. As shown in Fig. 1A, the flat bone has 2 hard walls, which were named the external table and the internal table. The hard walls are made of compact bone. The region between the 2 walls is called the diploe, which is composed of cancellous bone [31]. Compact bone has low porosity (5% to 10%) with interconnected tube-like pores, which are called Haversian canals and Volkmann canals [32]. The canals contain blood vessels ensuring the exchange of nutrients and metabolic wastes [1]. The cancellous bone stores bone marrow, and 80% of bony remodeling processes take place in this part [33]. The cancellous bone of the diploe is composed of irregular sponge-like trabeculae with a high specific surface area, and the mean surface curvature of the trabeculae surface is close to zero [34]. These architectural characteristics are similar to those of gyroids, which are representative triply periodic minimal surface structures. It was reported that the gyroid bioceramic scaffolds remarkably promoted in vitro and in vivo angiogenesis and bone regeneration [35,36].

Inspired by the composition and structural features of cranial bones, for the first time, we designed and fabricated 2 flat-bone-mimetic β-TCP bioceramic scaffolds, namely, Gyr-Comp scaffolds and Gyr-Tub scaffolds. Both scaffolds had gyroid pore structures in the inner layers. The Gyr-Comp scaffolds had 2 compact outer layers, which were designed according to the low-porosity feature of the outer tables of flat bones. The Gyr-Tub scaffolds had tubular pore structures in the 2 outer layers, which were designed to mimic the tube-like Haversian canals and Volkmann canals in the outer tables. The biomimetic architecture of bioceramic scaffolds was realized utilizing VPP-based 3D printing. We expected that the biomimetic scaffolds would facilitate angiogenesis and osteogenesis of cranial defects (Fig. 1). The pore morphology, mechanical strength, and cellular response were investigated in vitro. Rabbit cranial bone defect models were created to verify the promoting effects of the biomimetic structure of scaffolds on osteogenesis and angiogenesis. Furthermore, finite element analysis was conducted to elucidate the probable mechanism of the mechanical response and in vivo angiogenesis and osteogenesis of the biomimetic scaffolds. The flat-bone-mimetic scaffolds provide a promising treatment for cranial defect restoration.

## Results and Discussion

### Fabrication and characterization of flat-bone-mimetic β-TCP bioceramic scaffolds

**Fig. 2.**
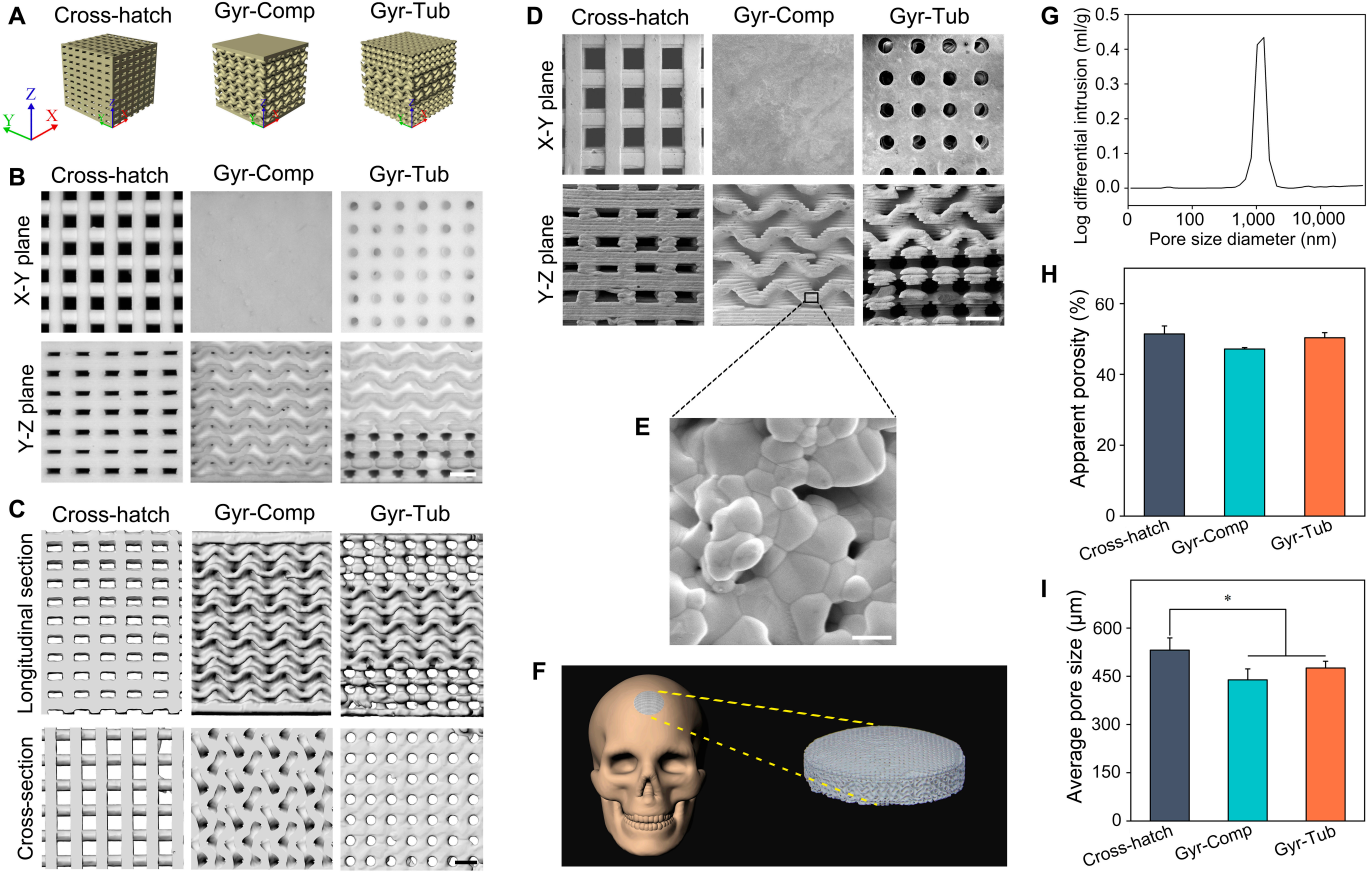
Design and fabrication of flat-bone-mimetic bioceramic scaffolds. (A) The designed models of Cross-hatch, Gyr-Comp, and Gyr-Tub scaffolds. (B) Stereomicrograph of the Cross-hatch, Gyr-Comp, and Gyr-Tub bioceramic scaffolds. Scale bar = 1 mm. (C) μ-CT photographs displaying the internal pore morphology of the scaffolds. Scale bar = 1 mm. (D) SEM photographs showing the detailed microstructure of the scaffolds. Scale bar = 1 mm. (E) High-magnification SEM images showing the micropores of the scaffolds. Scale bar = 2 μm. (F) The contour of flat-bone-mimetic scaffolds was designed for human cranial defect restoration. (G) Micropore size distribution in the bioceramic scaffolds. (H) Apparent porosity of the Cross-hatch, Gyr-Comp, and Gyr-Tub scaffolds (*n* = 4). (I) Average macropore size of the Cross-hatch, Gyr-Comp, and Gyr-Tub scaffolds (*n* = 12). The data are denoted as the mean ± SD. *P < 0.05 represents a significant difference.

We originally designed 2 kinds of flat-bone-mimetic structures, Gyr-Comp and Gyr-Tub (Fig. 2A). Gyr-Tub was composed of an inner layer with a gyroid pore structure and 2 outer layers with tubular pores. The proportion of inner and outer layers in Gyr-Tub was readily adjusted. The Gyr-Comp consisted of an inner layer and 2 compact outer layers. The gyroid pore structure in the inner layer of Gyr-Comp was identical to that of Gyr-Tub. To diminish the porosity difference between Gyr-Comp and Gyr-Tub, 2 thin outer layers were designed in Gyr-Comp, and the thickness ratio of the 2 compact layers to the gyroid porous layer was 1:9. The cross-hatch structure, which is the most conventional type of scaffold, was used as a control. The flat-bone-mimetic β-TCP bioceramic scaffolds were fabricated by VPP-based 3D printing and subsequent debinding and sintering. A single phase of β-Ca_3_(PO_4_)_2_ was identified in the biomimetic bioceramic scaffolds by the use of x-ray diffraction (Fig. S1). The presence of impurity elements (magnesium and strontium) in the synthesized β-TCP powders prevented the transformation of β-Ca_3_(PO_4_)_2_ to α- Ca_3_(PO_4_)_2_ at 1,200 °C [37]. A stereomicroscope was utilized to observe the general morphology of the scaffolds (Fig. 2B). The stereomicrographs indicated that the biomimetic bioceramic scaffolds well reproduced the pore morphology of the designed models. Both Gyr-Comp and Gyr-Tub showed curved surfaces of pores in their inner layers. Tubular pores were found in the 2 outer layers of Gyr-Tub scaffolds. Microcomputed tomography (μ-CT) images were indicative of the full interconnection between gyroid pores and tubular pores in the Gyr-Tub scaffolds (Fig. 2C and Fig. S2A). Scanning electron microscopy (SEM) was used to observe the details of the microstructure of the scaffolds (Fig. 2D). The interlayers were formed due to the layer-by-layer manufacturing manner of 3D printing. The gyroid porous layer was well integrated with the compact layers or tubular porous layers, free of separation between different structures. A few micropores were present in the pore walls of the scaffolds (Fig. 2E and Fig. S2B). Mercury intrusion porosimetry results showed that the micropores in the bioceramic scaffolds were sized at 0.5 to 4 μm (Fig. 2G). It has been well documented that the introduction of micropores into macroporous scaffolds is beneficial to bone generation and osteointegration [38]. The scaffold could be customized to match the contour of the human cranium (Fig. 2F). The apparent porosity of scaffolds was measured by the Archimedes method. The porosities of the Cross-hatch, Gyr-Comp, and Gyr-Tub scaffolds were 51.5%, 47.2%, and 50.4%, respectively (Fig. 2H). The lower porosity of the Gyr-Comp scaffolds was due to the presence of 2 compact outer layers. The average macropore sizes of Gyr-Tub, Gyr-Comp, and Cross-hatch were 469, 439, and 548 μm, respectively (Fig. 2I). Numerous studies have demonstrated that pores sized at 300 to 600 μm are favorable for the growth of new bone tissues and blood vessels [39,40]. The thickness of the dipole layer of the cranium varies with human sex and age [2]. We fabricated Gyr-Tub scaffolds with various thickness ratios of gyroid pores (Fig. S3), which could meet the needs of the patients.

### Mechanical response of flat-bone-mimetic β-TCP bioceramic scaffolds

**Fig. 3.**
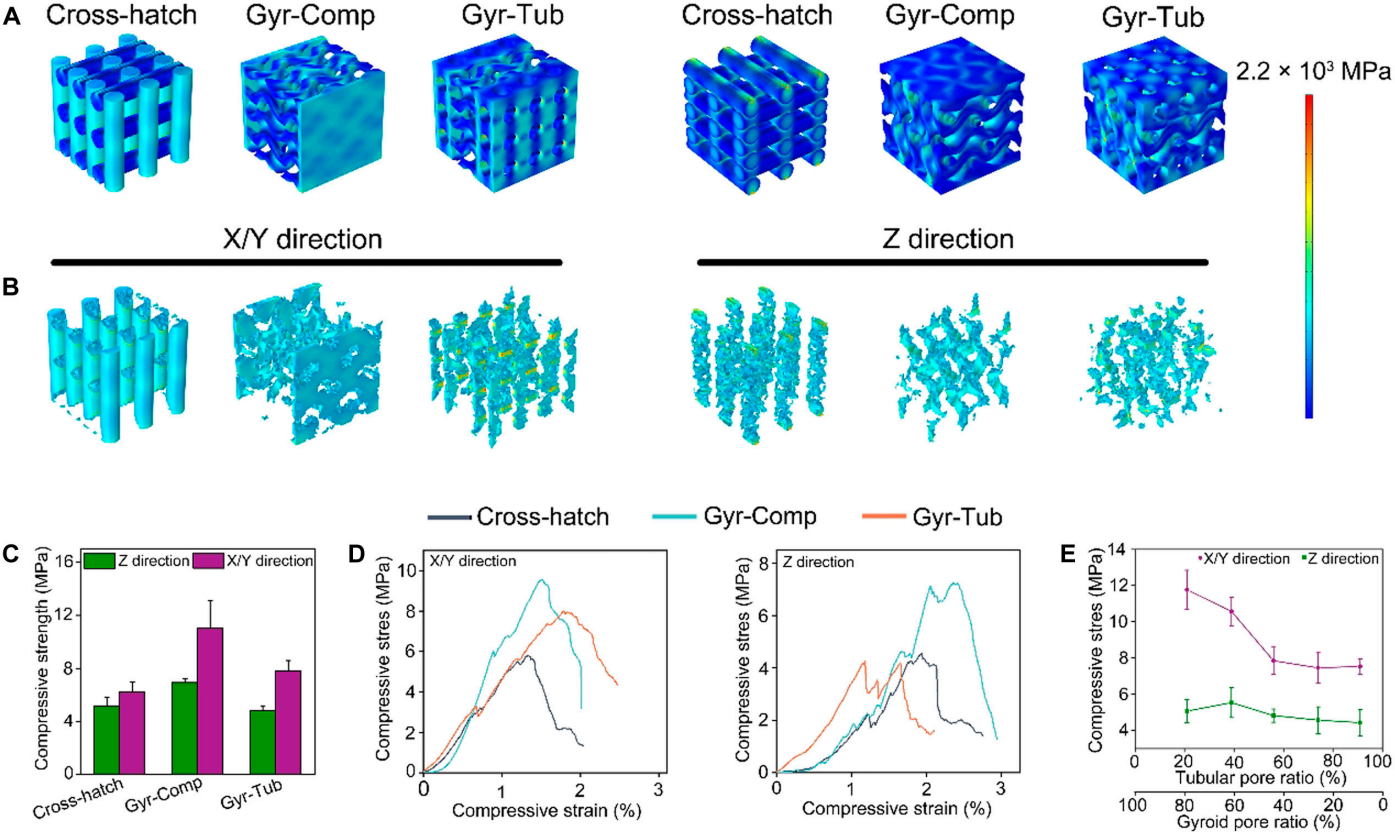
Mechanical behaviors of the flat-bone-mimetic scaffolds. (A) Finite element simulation showing von Mises stress distributions of the Cross-hatch, Gyr-Comp, and Gyr-Tub scaffolds with a compressive strain of 0.5%. The stress levels are indicated by the color scale. (B) The high-stress zones of the scaffolds. (C) Compressive strength of the scaffolds along the Z and X/Y directions. (D) Compressive stress–strain curves of the scaffolds in the Z and X/Y directions. (E) Compressive strength of the Gyr-Tub scaffolds with various thickness ratios of gyroid porous layer to tubular porous layer.

To investigate the effects of pore architecture on the mechanical behaviors of biomimetic bioceramic scaffolds, a finite element simulation was performed to show the stress distribution on the scaffold models with a compressive strain of 0.5% (Fig. 3A and B and Fig. S4). The maximum stress of the scaffolds with strain along the X or Y (X/Y) direction was lower than that along the Z direction; this demonstrated that Z-direction compression is more liable to cause cracks in the scaffolds than X/Y-direction compression. It was anticipated that the compressive strength of bioceramic scaffolds along the Z direction would be lower than that along the X/Y direction. The architectures of Gyr-Comp and Gyr-Tub were hybrids of 2 different structures. This explained why the maximum stress appeared at the interface junction between the 2 structures. The Cross-hatch scaffolds were found to have maximal stress at the junction between the intersecting filaments (Fig. S4). Notice that the Cross-hatch scaffolds had markedly more stress concentrations than the Cross-Comp and Gyr-Tub scaffolds (Fig. 3B). These results suggested that the Gyr-Comp and Gyr-Tub scaffolds had stronger resistance to breakage under compression.

To verify the computational results of the mechanical simulation, compressive tests were performed on the scaffold samples along the Z and X/Y directions. As shown in Fig. 3C, the compressive strength along the X/Y direction was noticeably higher than that along the Z direction for each group of scaffold samples, and the Cross-hatch scaffolds had the lowest compressive strength, which coincided with the results predicted by the finite element simulation. The finite element simulation showed that the maximal stress formed in the Gyr-Tub scaffolds was markedly higher than that in the Gyr-Comp scaffolds (Fig. S4); this suggested that the cracks were more prone to generate in the Gyr-Tub, accelerating the material damage under compression. In addition, the porosity of Gyr-Com scaffolds was slightly lower than that of Gyr-Tub scaffolds. Hence, the Gyr-Comp scaffolds had higher compressive strength in all directions than the Gyr-Tub scaffolds. Figure 3D displays the typical compressive stress–strain curves for the scaffolds loaded along the Z and X/Y directions. Unlike the nonporous block ceramics featuring sudden rupture and low deformation, the scaffolds were discovered to have strains as high as 3%, and the compressive strength did not linearly increase until final breakage. The presence of pores blunts the cracks and suppresses the crack propagation, leading to transformation from a sudden brittle rupture toward a diffuse breakage [41]. Even though the cracks were successively produced, the remaining crack-free part of scaffolds can bear the stress formed by the strains, thus leading to large compressive strains instead of catastrophic breakage [42]. The energy absorption was calculated by the stress–strain curves of scaffolds. The variation trend in energy absorption capacities for the scaffolds was similar to that in compressive strength (Fig. S5A). The variation in the proportion of tubular porous layers and gyroid porous layers did not alter the Z-direction compressive strength of Gyr-Tub scaffolds. The X/Y-direction compressive strength of Gyr-Tub scaffolds increased with an increasing proportion of the gyroid porous layer (Fig. 3E and Fig. S5B). For example, the Gyr-Tub scaffolds with 80% tubular layers had higher X/Y-direction compressive strength than the Gyr-Comp scaffolds, even though the Gyr-Comp scaffolds had a relatively lower porosity. In summary, the biomimetic scaffolds (Gyr-Comp and Gyr-Tub) had stronger resistance to compressive loading than the conventional Cross-hatch scaffolds, and Gyr-Comp had the best mechanical properties. Although the cranial bone is not a load-bearing tissue, the Gyr-Comp and Gyr-Tub scaffolds could protect the brain from external compression.

### Rat bone marrow mesenchymal stromal cell adhesion, proliferation, and osteogenic differentiation on the flat-bone-mimetic scaffolds

**Fig. 4.**
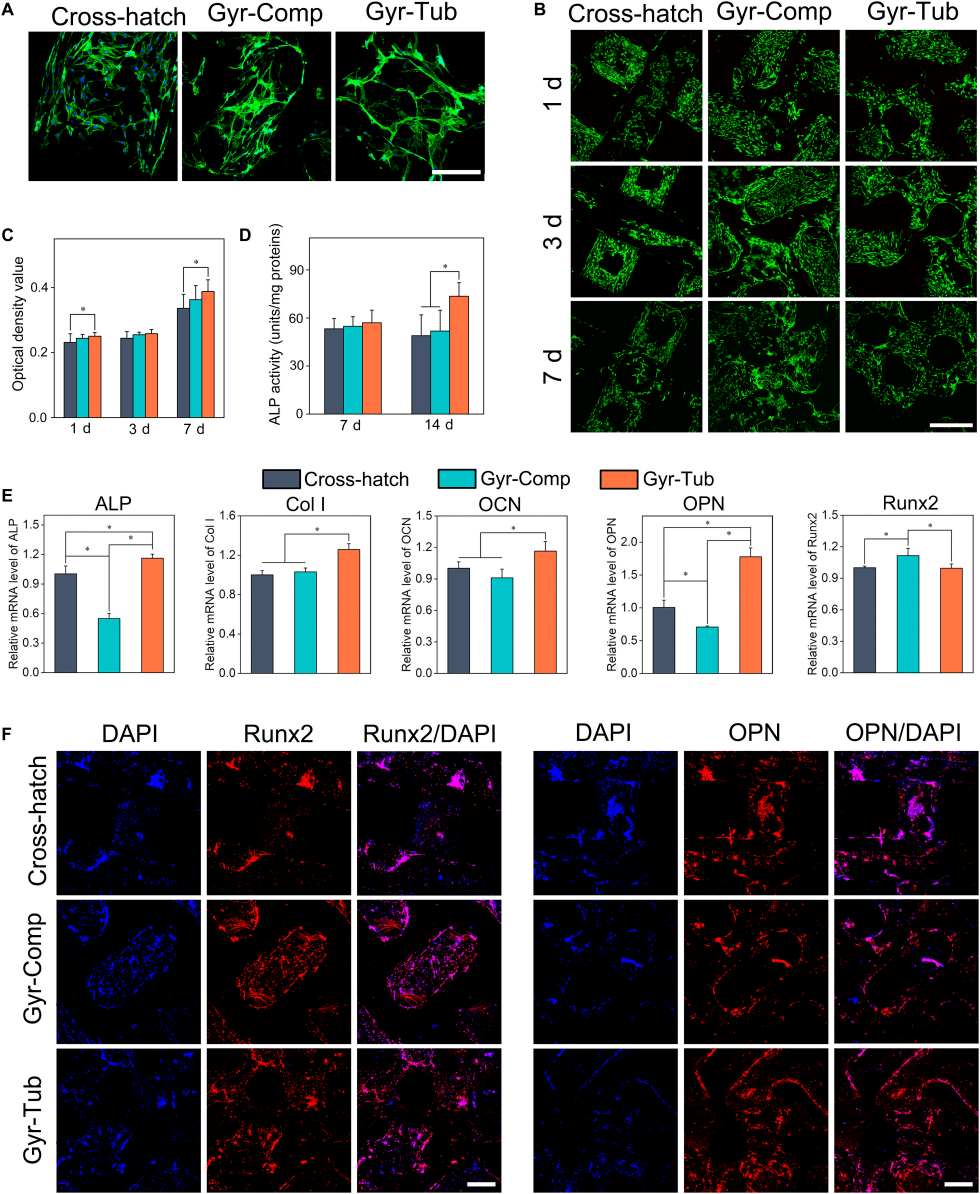
Adhesion, proliferation, and osteogenic differentiation of rBMSCs on biomimetic bioceramic scaffolds. (A) Morphology of rBMSCs adhering to the scaffolds. Scale bar = 500 μm. (B) Fluorescent photographs of live cells and dead cells on the scaffolds after culturing for 1, 3, and 7 d. Scale bar = 500 μm. (C) rBMSC proliferation on days 1, 3, and 7. (D) ALP activity of rBMSCs on the 7th and 14th days. (E) mRNA expression of osteogenesis-related genes in rBMSCs on the seventh day. (F) Immunofluorescence staining of osteogenic proteins expressed in the rBMSCs after 7 d of culture. Scale bar = 300 μm. The data are denoted as the mean ± SD. *P < 0.05 represents a significant difference (*n* = 4).

Cell adhesion and proliferation are critical performance indicators of bone regenerative scaffolds [43]. Immunofluorescence staining of cytoskeletal F-actin was conducted, and the morphology of rat bone marrow mesenchymal stromal cells (rBMSCs) adhering to the scaffolds was observed employing a laser confocal microscope (Fig. 4A). After 24 h of culturing, the rBMSCs seeded on the scaffolds were well spread and elongated. The viability and proliferation of rBMSCs on the scaffolds were qualitatively evaluated by the Live/Dead assay (Fig. 4B and Fig. S6). Many live cells (green fluorescence) were distributed on the surfaces of all the scaffolds, and very few dead cells (red fluorescence) could be seen. There were more live cells growing on the scaffolds as the culture time was prolonged. Cell proliferation was quantitatively assessed by the CCK-8 kit (Fig. 4C). The rBMSCs seeded on the scaffolds progressively proliferated over time. It seemed that the cell proliferation of the Cross-hatch group was inferior to that of the Gyr-Comp and Gyr-Tub groups, and the Gyr-Tub groups had the best cell proliferation. However, statistical analysis results indicated that on the first and third days, there was no obvious difference between the 3 scaffolds. On the seventh day, the cell number in the Gyr-Tub group was significantly larger than that in the Cross-hatch group, but no statistically significant difference was found between the Gyr-Tub and Gyr-Comp groups.

Alkaline phosphatase (ALP) is a vital marker of early-stage osteogenic differentiation [44]. ALP activity was quantitatively determined by the p-nitrophenyl phosphate assay (Fig. 4D). After culturing for 7 d, the ALP activity of rBMSCs was at the same level for the 3 scaffolds. As the culture time extended to 14 d, the Gyr-Tub group showed the highest ALP activity, while the Gyr-Comp group was comparable to the Cross-hatch group. Photographs of ALP staining showed that only a small amount of ALP formed on all the scaffolds after 7 d of cell culture (Fig. S7). On the 14th day, the ALP staining density of the Gyr-Tub group was markedly higher than that of the Cross-hatch and Gyr-Comp groups. Calcium nodules are a typical marker of late-stage osteogenic differentiation [45]. Alizarin Red S staining was utilized to evaluate the formation of calcium nodules in rBMSCs, which were cultured on the scaffolds for 14 d (Fig. S8). Substantial calcium nodules were deposited on all the scaffolds, indicating the positive effects of the scaffolds on the mineralization of rBMSCs. Real-time polymerase chain reaction was employed to assess the expression of osteogenesis-associated genes (ALP, osteocalcin [OCN], osteopontin [OPN], collagen type I [Col I],and runt‐related transcription factor 2 [Runx2]) in rBMSCs cultivated on biomimetic scaffolds for 7 d (Fig. 4E). OCN and OPN are closely related to late-stage osteogenesis and biomineralization [46]. Runx2 plays crucial roles in multiple steps of bone development. Runx2 affects the differentiation of mesenchymal stem cells into the osteoblast lineage and promotes osteoblastic differentiation at the early stage [47]. The Gyr-Tub group showed the highest mRNA expression of ALP, Col I, OCN, and OPN. The Gyr-Comp group expressed the highest level of Runx2, and the Runx2 expression in the Gyr-Tub group was comparable to that in the Cross-hatch group. The Col I and OCN expression levels in the Gyr-Comp group and Cross-hatch group were similar. The Cross-hatch group showed the lowest mRNA expression of ALP and OPN. Immunofluorescence staining was used to assess the osteogenic protein expression of rBMSCs cocultured with the biomimetic scaffolds (Fig. 4F). All scaffolds stimulated the secretion of OPN and Runx2 after culturing for 7 d. Semiquantitative analysis showed that Gyr-Tub was most effective in promoting the production of OPN. Gyr-Comp and Gyr-Tub markedly enhanced the expression of Runx2, in contrast with the Cross-hatch group (Fig. S9). On the whole, the Gyr-Tub scaffolds were most favorable for rBMSC proliferation and osteogenic differentiation.

### Human umbilical vein endothelial cell proliferation and angiogenesis on flat-bone-mimetic scaffolds

**Fig. 5.**
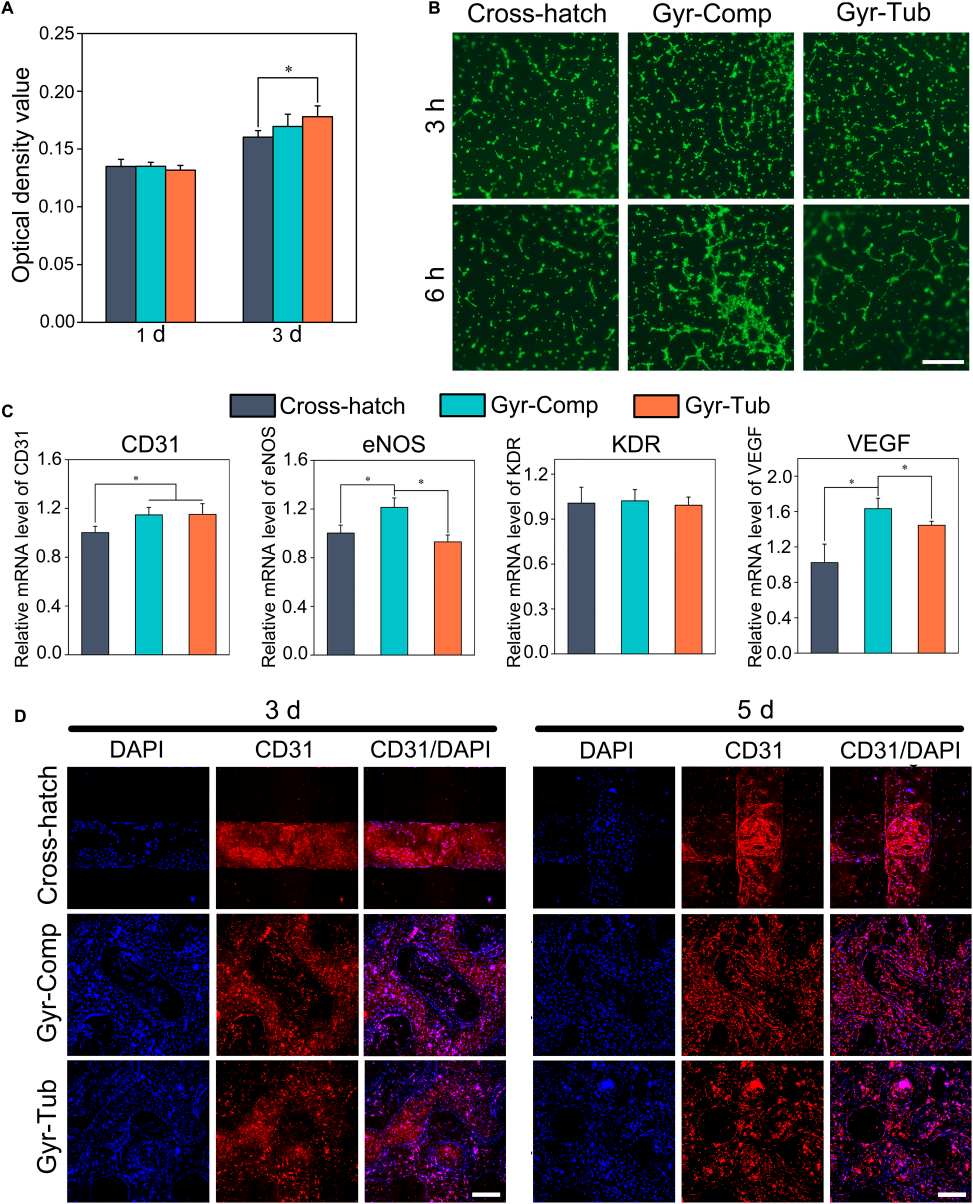
Proliferation and angiogenic potential of HUVECs cultured on biomimetic bioceramic scaffolds. (A) Proliferation of HUVECs cultivated on the scaffolds. (B) Calcein-acetoxymethyl staining images showing the tube formation of HUVECs after incubation for 3 and 6 h. Scale bar = 500 μm. (C) Angiogenesis-related gene expression in HUVECs on day 3. (D) Immunofluorescence staining of angiogenic proteins expressed in HUVECs after 3 and 5 d of cell culture. Scale bar = 300 μm. The data are denoted as the mean ± SD. *P < 0.05 represents a significant difference (*n* = 4).

A live/dead assay showed that many viable cells attached to the scaffolds, and the number of viable cells increased over time (Fig. S10). The quantitative results of cell proliferation evaluated by CCK-8 assay were in good agreement with the information given by the Live/Dead assay (Fig. 5A). On day 1, there was no obvious difference in cell numbers between the 3 groups. As the culture time was prolonged to 3 d, Gyr-Tub showed better cell proliferation than the Cross-hatch scaffolds, while there was no distinct difference between the 2 biomimetic scaffolds. Tubule formation by endothelial cells is a pivotal process in angiogenesis [48]. The angiogenic potential of human umbilical vein endothelial cells (HUVECs) cultured on the scaffolds was evaluated by the tubule formation assay (Fig. 5B). After incubation for 3 h, all the groups did not show an apparent tubular network but substantial discontinuous tubule walls. After 6 h of incubation, the Gyr-Tub and Gyr-Comp groups generated capillary-like networks, while the Cross-hatch group only formed plentiful vascular branches and discontinuous tubule walls. Real-time polymerase chain reaction was used to investigate the mRNA expression of angiogenic genes (platelet endothelial cell adhesion molecule-1 [CD31], endothelial nitric oxide synthase [eNOS], vascular endothelial growth factor receptor 2 [KDR], and vascular endothelial growth factor [VEGF]) in HUVECs cultivated on the scaffolds for 3 d (Fig. 5C). CD31 is a well-documented sensitive and specific marker for the differentiation of vascular endothelial cells [49]. The Gyr-Comp and Gyr-Tub groups expressed higher levels of CD31 than the Cross-hatch group. eNOS is responsible for the production of NO, which maintains vascular homeostasis [50]. The Gyr-Comp group showed the highest mRNA expression of eNOS and VEGF. The eNOS expression of the Gyr-Tub group was similar to that of the Cross-hatch group. The VEGF expression level in the Gyr-Tub group was higher than that in the Cross-hatch group. There was no obvious difference in KDR expression between the 3 groups. Immunofluorescence staining of CD31 was conducted to further evaluate the angiogenic function of HUVECs cultured on the scaffolds for 3 and 5 d (Fig. 5D). The nuclei and CD31 were stained blue and red, respectively. Semiquantitative analysis showed that the Gyr-Tub and Gyr-Comp groups showed more intense CD31 fluorescence than the Cross-hatch group (Fig. S11). Taken together, the flat-bone-mimetic scaffolds (Gyr-Com and Gyr-Tub) were more beneficial to HUVEC proliferation and angiogenesis than the conventional Cross-hatch scaffolds.

### In vivo bone regeneration evaluation of the flat-bone-mimetic scaffolds

**Fig. 6.**
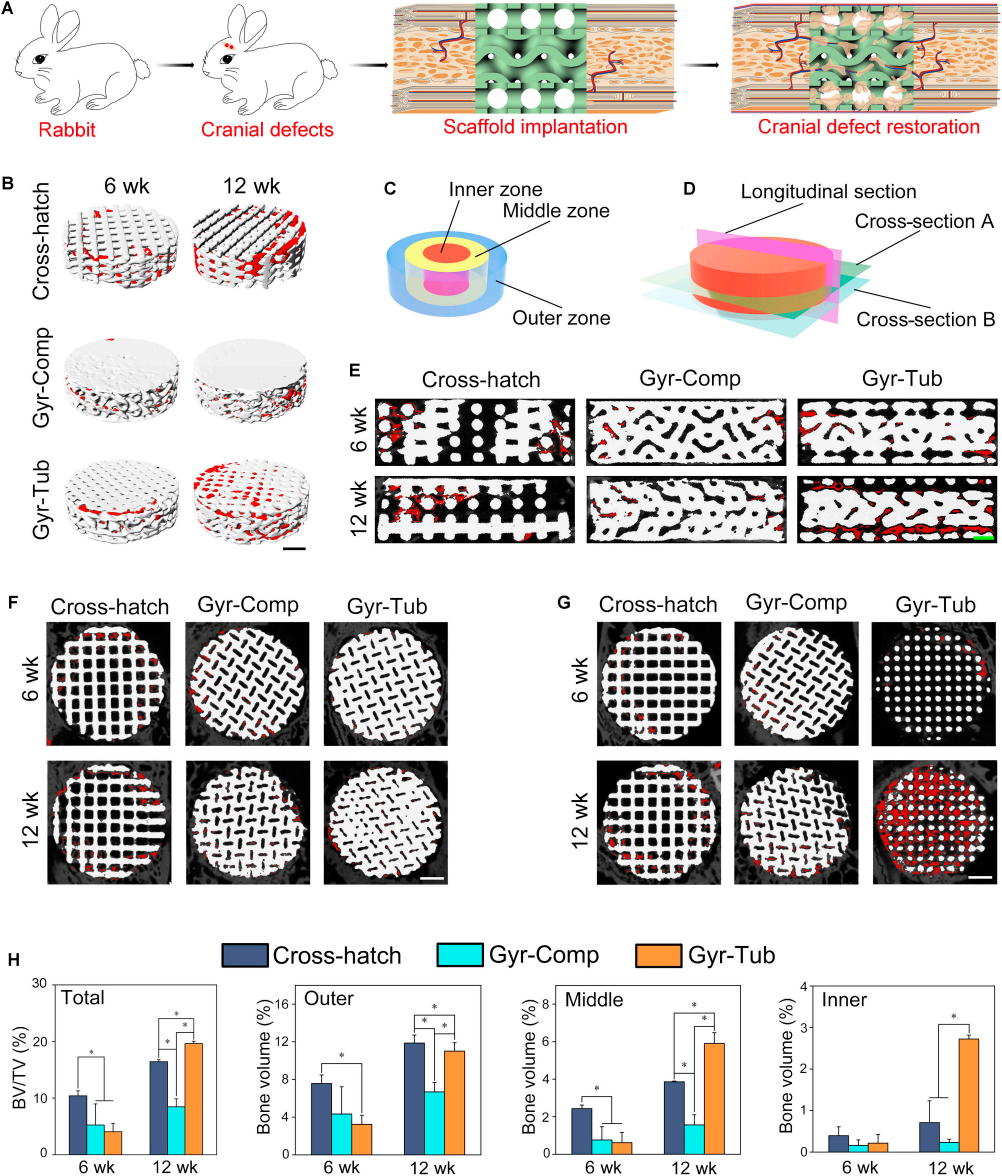
In vivo bone generation evaluation of the biomimetic scaffolds implanted into rabbit cranial defects. (A) Schematic illustration of the animal experiment to evaluate the effects of flat-bone-mimetic scaffolds on rabbit cranial defect restoration. (B) 3D reconstructed μ-CT photographs of the scaffold implants at postoperative weeks 6 and 12. Scale bar = 2 mm. (C) Schematic diagram of the outer, middle, and inner zones of the scaffold implants. (D) Schematic diagram of 3 sections for μ-CT photographs. Cross-section A denotes the cross-section at the one-half height of the scaffolds. Cross-section B denotes the cross-section at the one-fourth height of the scaffolds. (E) μ-CT photographs of longitudinal sections of the scaffold implants at 6 and 12 weeks postoperation. Scale bar = 1 mm. (F) μ-CT photographs of cross-section A. Scale bar = 2 mm. (G) μ-CT photographs of cross-section B. Scale bar = 2 mm. (H) Total bone volume fraction and the bone volume percentage in the outer, middle, and inner zones of scaffold implants at 6 and 12 weeks postsurgery (*n* = 4). Data are denoted as the mean ± SD. *P < 0.05 represents a significant difference.

Rabbit cranial defect models were developed to investigate the effect of flat-bone-mimetic bioceramic scaffolds on the bone regeneration of cranial defects (Fig. 6A and Fig. S12). At 6 and 12 weeks postoperation, the samples were harvested to test the osteogenesis and angiogenesis of the scaffold implants. μ-CT analysis was employed to assess the bone generation of the cranial defects implanted with various scaffolds (Fig. 6B to H). The scaffolds and newly generated bone tissues were rendered in white and red, respectively. The 3D reconstructed μ-CT photographs show that the bone tissues grew into the pores of the scaffolds, and the peripheral region of the scaffolds was filled with substantial newly formed bone tissues (Fig. 6B). To investigate in detail the effects of the pore architecture of scaffolds on bone regeneration, μ-CT photographs of various sections of cylindrical scaffold implants are presented in Fig. 6D to G. Figure 6E shows μ-CT photographs of longitudinal sections of implants. At postoperative week 6, the events of bone regeneration occurred mainly in the zones close to the surface of all the scaffolds, and the Cross-hatch group was found to have more bone regeneration than the Gyr-Comp and Gyr-Tub groups. At the 12th week, more bone regeneration was found in all the scaffold implants. The bone regeneration inside the Gyr-Comp group was inferior to that inside the Cross-hatch group. It is worth noting that the bone regenerated throughout the whole Gyr-Tub scaffold implants. The μ-CT photographs of cross-sections at one-half height of implants could display the bone generation in the gyroid porous layer of Gyr-Tub scaffolds, and those at one-fourth height of implants could present the bone generation in the tubular porous layer of Gyr-Tub scaffolds and the gyroid porous layer of Gyr-Comp scaffolds (Fig. 6D, F, and G). At 6 weeks after implantation, the new bone mainly accumulated in the zones close to the surface of all the scaffold implants. The Cross-hatch group showed the most newly formed bones, whereas the Gyr-Comp and Gyr-Tub groups displayed more bone formation in the zones close to the center of gyroid pores. This suggested that the gyroid pores are conducive to the inward growth of bone tissues. The new bone generation in the tubular pores of Gyr-Tub seemed inferior to that in the gyroid pores of Gyr-Comp and square-like pores of the Cross-hatch group. At postoperative week 12, despite having more bone formation, the Cross-hatch and Gyr-Comp groups did not show significantly more bone generation in the zones close to the center of the scaffolds, and the bone tissues were predominantly generated in the regions close to the surfaces of scaffolds. In contrast, the bone tissues regenerated throughout the tubular pores of Gyr-Tub scaffolds. All scaffold implants barely degraded in the implantation period. The bone volume fraction (BV/TV) reflects the bone volume change [51]. To investigate the bone ingrowth effects of the scaffolds, the scaffold implants were divided into outer, middle, and inner zones (Fig. 6C), and the bone formation volume fraction in different zones was quantitatively evaluated by μ-CT (Fig. 6H). At the sixth week, the Cross-hatch group had the highest bone formation volume, while there was no distinct difference between the 2 flat-bone-mimetic scaffolds. The Cross-hatch group showed the highest bone formation fraction in the outer and middle zones. The bone formation volume percentage in the inner zone of the 3 scaffolds was lower than 0.5%. At postoperative week 12, the Gyr-Tub group outperformed the Cross-hatch group in bone generation percentage. Gyr-Comp had the lowest bone generation percentage. The tremendous increase in bone formation of Gyr-Tub scaffolds was contributed by the noticeably increased bone generation in the middle and inner zones. Bone formation in the inner zone of the Cross-hatch group was not profoundly increased.

**Fig. 7.**
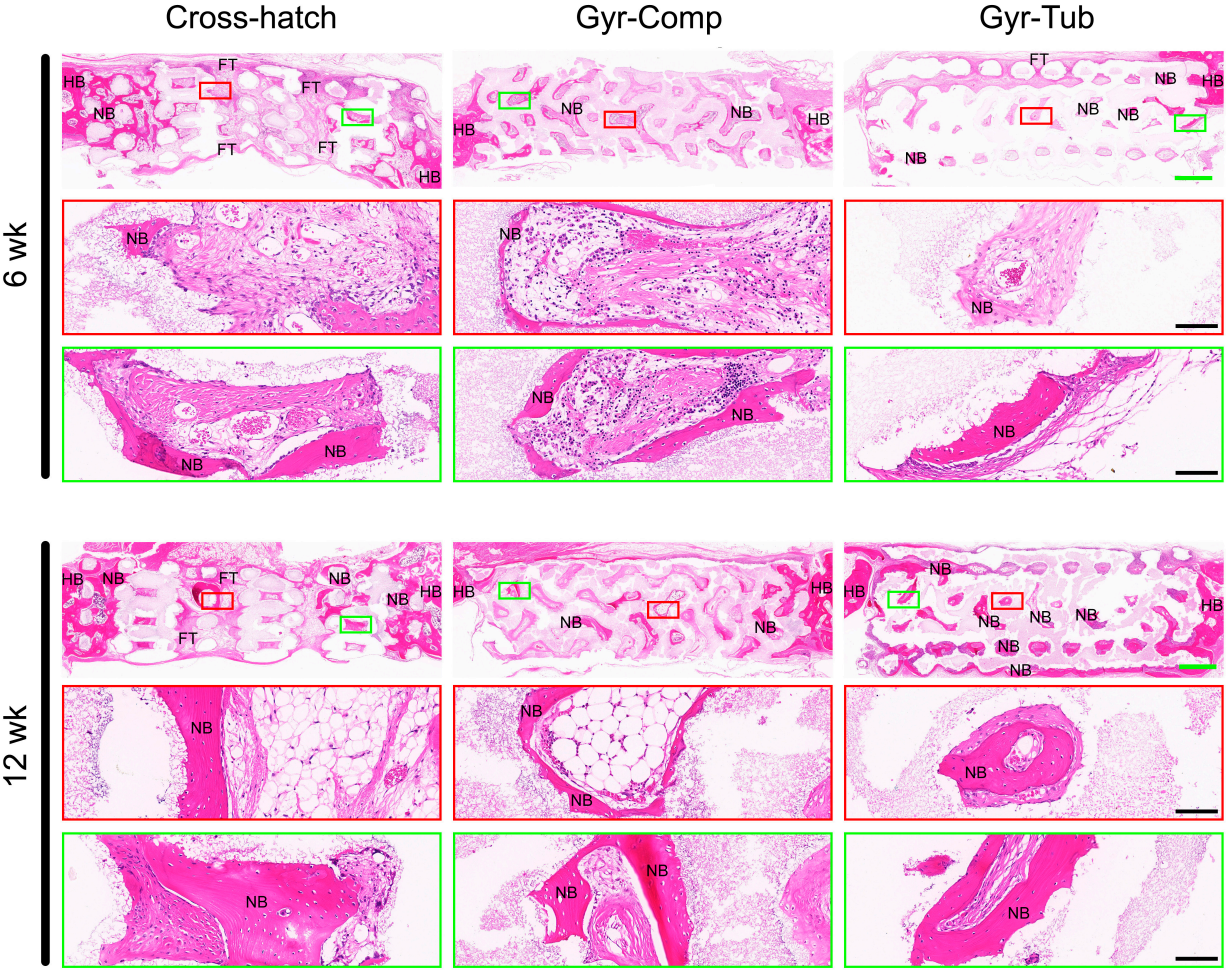
Histological examinations of HE staining of rabbit cranial defects after implantation for 6 and 12 weeks. Green scale bar = 1 mm; black scale bar = 200 μm. NB, new bone; FT, fibrous tissue; HB, hosting bone.

Histological examinations by hematoxylin and eosin (HE) staining and Masson’s staining were conducted to further verify the effects of flat-bone-mimetic scaffolds on repairing cranial defects (Fig. 7). As shown in the photographs of HE-stained samples, at 6 weeks postimplantation, all the scaffold implants had substantial new bone tissues in the regions close to the host bones. Apparently, the Cross-hatch implants showed the most new bone tissues, but very few new bone tissues were found in the inner region. In contrast, considerable amounts of thin bone trabeculae were found in the interior of the Gyr-Comp and Gyr-Tub scaffolds. Of note, more fibrous tissues grew inward the pores of Gyr-Tub and Cross-hatch implants compared with the Gyr-Comp implants. At postoperative week 12, more bone tissues regenerated in the pores of all the scaffold implants. However, the pores in the inner zones of Cross-hatch implants were still mainly occupied by fibrous tissues, with very few bone tissues observed. In contrast, the new bone tissues in the inner regions of the 2 flat-bone-mimetic scaffolds profoundly increased, and the Gyr-Tub showed markedly more bone tissues than the Gyr-Comp group. Overall, the bone regeneration results evaluated by HE staining were consistent with those evaluated by μ-CT (Fig. 6B to H). Masson’s staining was performed to evaluate the mineralization degree of new bone in the scaffold implants. The new bone tissues in all scaffolds were dominated by woven bone with low mineralization. There was no distinct difference in mineralization degree between the 3 groups (Figs. S13 and S14).

**Fig. 8.**
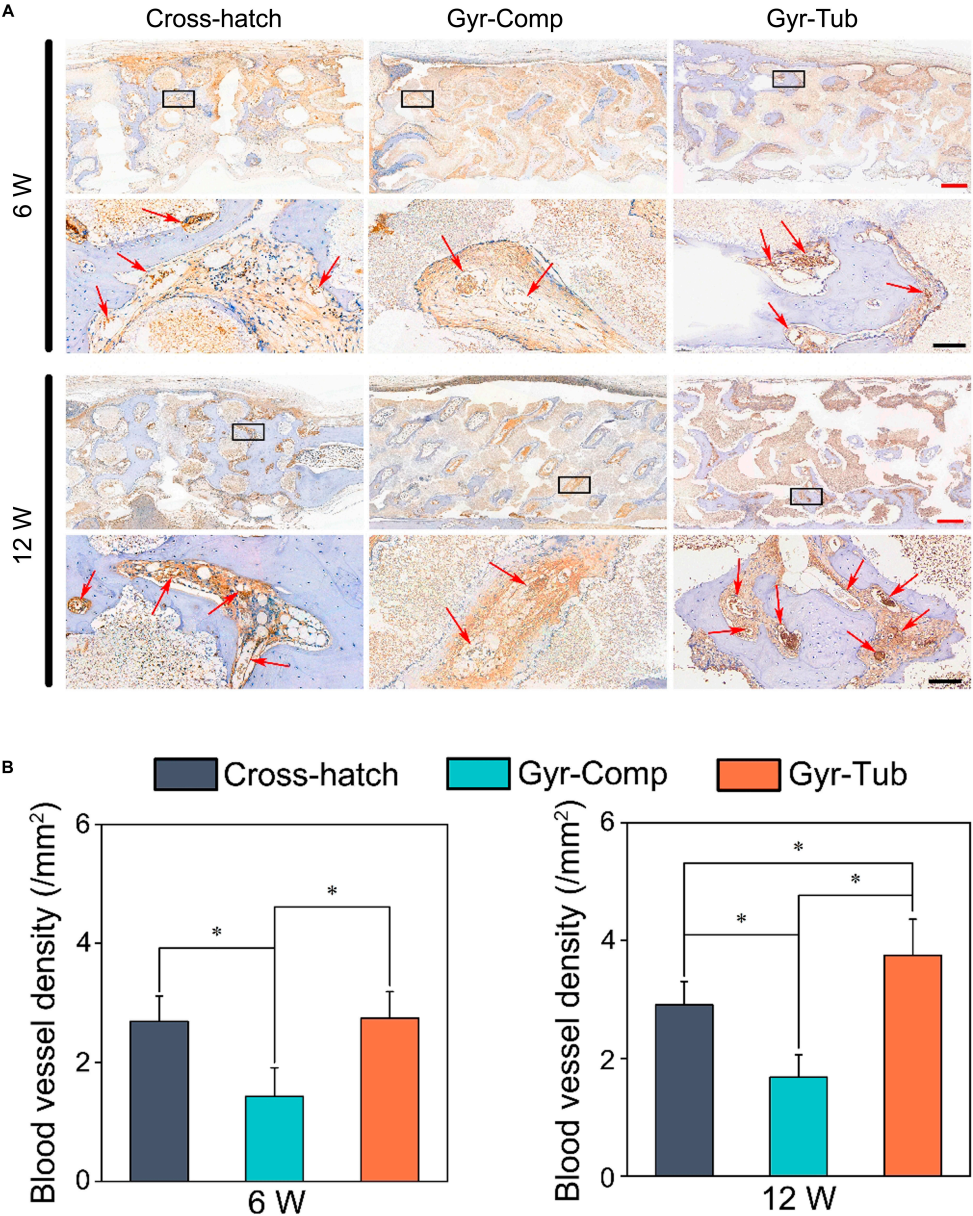
(A) vWF immunohistochemical examination. (A) vWF immunohistochemical photographs of rabbit cranial defects implanted with scaffolds. Red scale bar = 500 μm, black scale bar = 200 μm. Red arrows point to the vWF-positive blood vessels. (B) Quantitative analysis of vWF-positive blood vessels after implantation for 6 and 12 weeks. Data are denoted as the mean ± SD. *P < 0.05 represents a significant difference (*n* = 4).

Immunohistochemical staining of von Willebrand factor (vWF) and CD31 was conducted to assess the angiogenesis of scaffolds in vivo (Fig. 8 and Fig. S15). vWF is characterized by selective endothelial expression, so vWF together with CD31 has been extensively used to evaluate in vivo angiogenesis [52]. At 6 weeks after the operation, the Gyr-Comp group had a significantly smaller number of blood vessels than the other 2 groups, and the number of blood vessels in the Gyr-Tub group was comparable to that in the Cross-hatch group. At the 12th week, the Gyr-Tub group had the greatest amount of blood vessels, followed by the Cross-hatch group. Both the Gyr-Comp and Cross-hatch groups did not attain more blood vessel generation compared to that at postoperative week 6.

The results of in vivo investigations, including μ-CT scanning and HE, Masson’s, and immunohistochemical staining, showed that the Cross-hatch scaffolds were more effective in osteogenesis and angiogenesis in the early stage of implantation (6 wk), while Gyr-Tub outperformed Cross-hatch scaffolds in the generation of bone tissues and blood vessels in the later stage of implantation (12 wk). Gyr-Comp showed the weakest osteogenesis and angiogenesis capacities throughout the implantation period. The results of in vivo osteogenesis and angiogenesis were not consistent with those of the in vitro study. Compared with the conventional Cross-hatch scaffolds, the flat-bone-mimetic scaffolds (Gyr-Tub and Gyr-Comp) showed more favorable in vitro osteogenic and angiogenic performances, which were demonstrated by promoting rBMSC proliferation and osteogenic differentiation, as well as HUVEC proliferation and angiogenesis. Compared with Gyr-Comp scaffolds, Gyr-Tub showed a similar level of angiogenic potential but better in vitro osteogenic performance. In the cell experiments, the cells were seeded onto the surface of the scaffold. The in vitro cell response mainly hinges on the pore topology of scaffolds, due to their same composition and similar porosity. The tubular pores featured concave surfaces, which have been proven to be more favorable for the generation of bone tissues and blood vessels, in contrast with pores with flat or convex surfaces [23,24,52–55]. The gyroid pores have curved, hyperboloidal surfaces and numerous corners, which facilitate osteogenic differentiation of bone mesenchymal stem cells as well as proliferation and angiogenic differentiation of endothelial cells [21,28,35,36]. The gyroid and tubular pore structures were responsible for the enhancement of in vitro cell proliferation, osteogenesis, and angiogenesis of the Gyr-Comp and Gyr-Tub scaffolds. In vivo osteogenesis and angiogenesis are affected by the permeability of implants [56,57]. High permeability allows for more effective transportation of nutrients and oxygen inside the scaffolds, thus facilitating penetration of cells and tissues into the scaffolds [56,57]. Fluid dynamic simulations were conducted to assess the permeability of the scaffolds (Fig. S16 and Table S1). The permeability of Cross-hatch in all directions was higher than that of the Gyr-Comp and Gyr-Tub scaffolds. The higher permeability of Cross-hatch scaffolds may be responsible for their higher efficiency in the generation of bone tissues and blood vessels in early-stage implantation. With the extension of implantation time, Gyr-Tub achieved the best performances of angiogenesis and osteogenesis, owing to the beneficial biological effects of tubular pores and gyroid pores. The curved lateral surface of scaffolds was in contact with the hosting bones, while the 2 circular bases were in contact with the soft tissues. There are 2 sides to the soft tissues. On the one hand, fast ingrowth of soft tissues may obstruct new bone formation, leading to bone nonunion [58]. To repair bone defects surrounded by a large area of soft tissues, barrier membranes in conjunction with bone grafts can prevent fast invasion of soft tissues and simultaneously guide bone formation [59,60]. On the other hand, soft tissues are rich in blood vessels, so the ingrowth of soft tissues in early-stage implantation can accelerate the vascularization of scaffolds [61]. Histological and immunohistological assessments demonstrated that the presence of compact layers in Gyr-Comp scaffolds prevented fluid flow and ingrowth of blood vessel-rich soft tissues, leading to inferior angiogenesis and consequently poorer osteogenesis throughout the implantation period. Despite having the best mechanical performance, the weak bone regenerative capacity of Gyr-Comp makes them unsuitable for cranial defect restoration. The Gyr-Tub scaffolds also possessed good mechanical strength to protect the brain from external compression. Moreover, the Gyr-Tub scaffolds ideally mimicked the porous structure of the inner and external tables and diploe of the flat bone, which provides a biomimetic 3D structural environment for mesenchymal stromal cell adhesion, proliferation, and osteogenic differentiation, as well as endothelial cell proliferation and angiogenesis. Our future research will focus on further optimization of the architecture of Gyr-Tub scaffolds, and evaluations of vascularization and long-term material degradation and bone regeneration of Gyr-Tub scaffolds using the cranial defect models of large animals.

## Conclusion

In this study, to develop an alternative to autologous bone grafts for cranial defect reconstruction, 2 flat-bone-mimetic β-TCP bioceramic scaffolds (Gyr-Comp and Gyr-Tub) were designed and fabricated by VPP-based 3D printing. Gyr-Comp had 2 compact outer layers and a diploe-like inner layer with a gyroid pore structure. The Gyr-Tub consisted of 2 outer layers with tubular pores and a diploe-like inner layer. The anisotropic flat-bone-mimetic scaffolds differed in compressive strength along different directions. Compared with the conventional Cross-hatch scaffolds, the flat-bone-mimetic scaffolds possessed higher compressive strength and energy absorption capacity, and the Gyr-Comp scaffolds had the highest values. Gyr-Tub promoted in vitro cell proliferation, osteogenesis, and angiogenesis. Gyr-Comp presented high in vitro angiogenic activities. In the early stage of implantation (6 wk), the bone regeneration of rabbit cranial defects implanted with biomimetic scaffolds was inferior to that of defects implanted with Cross-hatch scaffolds, due to the higher permeability of Cross-hatch scaffolds. With the extension of implantation time (12 wk), Gyr-Tub outperformed Cross-hatch in the repairing effects of cranial defects, reflected by profoundly enhanced osteogenesis and angiogenesis. The Gyr-Comp scaffolds were the least effective implants for repairing cranial defects, because the presence of compact layers suppressed blood vessel ingrowth and decreased permeability. This study provides a novel concept for developing biomimetic bone regenerative materials, and Gyr-Tub scaffolds with favorable mechanical properties and excellent osteogenic and angiogenic capacities have high prospects for treating cranial bone defects in clinical applications.

## Materials and Methods

The detailed experimental procedures are presented in the Supplementary Materials. All animal experiments were approved by the Laboratory Animal Ethics Committee of Guangzhou Huateng Biomedical Technology, approval number: HTSW220418.

## Data Availability

The data relevant to this article are available from the corresponding author upon request.
