# Supplementary material for: 3D-Printed Flat-Bone-Mimetic Bioceramic Scaffolds for Cranial Restoration
Source: Research (Wash D C). 2023 Oct 26;6:0255. doi: 10.34133/research.0255 (PMC10603392; doi:10.34133/research.0255)
Supplement: Supplementary 1 — Materials and Methods Figs. S1 to S17 Tables S1 to S3 [file research.0255.f1.docx]

Supporting Information

**3D-printed flat-bone-mimetic bioceramic scaffolds for cranial restoration**

Yihang Zhang^1^**^†^**, Fupo He^1^**^†^**^*^, Qiang Zhang^2^**^†^**, Haotian Lu^3^, Shengtao Yan^3,4*^, Xuetao Shi^2*^

^1^School of Electromechanical Engineering, Guangdong University of Technology, Guangzhou 510006, P. R. China. ^2^School of Materials Science and Engineering, South China University of Technology, Guangzhou 510641, P. R. China. ^3^Peking Union Medical College Graduate School, Beijing 100730, P. R. China. ^4^Department of Emergency, Chian-Japan Friendship Hospital, Beijing 100029, P. R. China.

*Address correspondence to: shxt@scut.edu.cn (X.S.); [fphe@gdut.edu.cn](mailto:fphe@gdut.edu.cn) (F.H.); [yanshengtao@zryhyy.com.cn](mailto:yanshengtao@zryhyy.com.cn) (S.Y.)

†These authors contributed equally to this work

**Materials and methods**

**Preparation of biomimetic β-TCP bioceramic scaffolds**

The β-TCP powders were prepared by the wet-chemical method [1]. The liquid premixtures were prepared by manual mixing of 1,6-hexanediol diacrylate (HDDA), trimethylolpropane tri-acrylate (TMPTA), KOS110 dispersants, diphenyl (2,4,6-trimethyl benzoyl) phosphine oxide (TPO) photoinitiator and n-caprylic alcohol (defoaming agent). The weight ratios of HDDA, TMPTA, KOS110, TPO and n-caprylic alcohol were 40, 40, 5, 3, and 12 wt.%, respectively. The β-TCP powders were dispersed in the premixed liquid and then subjected to ball milling with a planetary ball mill (QM-2SP20, Nanjing University Instrument Factory, China) for 24 hours to acquire homogeneous photocurable pastes. Absolute ethyl alcohol was used as the dispersion medium, and the rotational speed was 245 rpm. The β-TCP solid content in the pastes was 43.1 vol.%.

The 3D models of the cross-hatch structure, solid layer, gyroid pore structure and tubular pore structure were established using Rhinoceros software (Robert McNeel & Assoc, USA). The gyroid pore structure was designed by an implicit equation, as described previously [2]. The files were imported into Magics software (Materialise, Belgium), and the gyroid pore structure was integrated with the tubular pore structure to form the Gyr-Tub scaffold model. The solid layer and gyroid pore structure were integrated to form the Gyr-Comp scaffold model. The models were trimmed and repaired. The document of the virtual printing platform was imported into Magics software. The designed scaffold models were arranged on the printing platform, and the files were exported in STL format.

The pore structures of scaffolds were manufactured by a VPP-based printer (Ceramaker 300 system, 3D CERAM, France), following the instructions presented in the previous work [2]. The pastes were solidified into green parts layer by layer after being exposed to an ultraviolet laser (wavelength: 365 nm; exposure intensity: 52 mW/cm^2^). The layer thickness was set to 100 μm. After the printing process, the green bodies of scaffolds were cleaned with water and ethanol, followed by air-drying (60 ℃), debinding and sintering (maximum temperature: 1200 ℃; heating rate: 3 ℃/min; dwell time: 1 h). The Cross-hatch, Gyr-Comp and Gyr-Tub bioceramic scaffolds were obtained for physicochemical characterizations, cell response assessments and in vivo implantation (**Fig. S17**).

**Scaffold characterizations**

Phase identification of the bioceramic scaffolds was conducted by X-ray diffraction (XRD; Empyrean PANalytical B.V., Netherlands) with Cu-Kα radiation. The voltage and current were 40 kV and 40 mA, respectively. The data were acquired in the range of 10°-70° for 2θ. The scanning step size was 0.01313°. The general morphology of the β-TCP scaffolds was observed using a stereomicroscope (SteREO Discovery. V12, Zeiss, Germany). The internal macroporous structure of the scaffolds was observed with microcomputed tomography (μ-CT; Harrier xtv160, Japan). Details of macropores and micropores were observed utilizing a scanning electron microscope (SEM; Q25, FEI, USA). The average pore size of the scaffolds was determined by size measurement of at least 12 macropores at various sites. The apparent porosity of the scaffolds was measured in accordance with the protocols provided by ISO 18754:2020, which is based on the Archimedes method. Absolute ethyl alcohol was used as the displacement liquid [1]. The size distribution of micropores in the scaffolds was tested with a mercury intrusion porosimeter (AutoPore V 9600, Micromeritics, USA). A penetrometer with a stem volume of 3.4669 mL was utilized. The scaffold samples were pressurized from 0.1 to 61,000 psi, and the mercury contact angle was 130°. The compressive strength of scaffolds (8 mm × 8 mm × 8 mm) was measured by a universal material testing machine (Instron 5967, Instron, USA). The crosshead rate was 0.5 mm min^-1^.

**Finite element analysis**

Simulations of quasistatic compression tests and computational fluid dynamics (CFD) were performed with finite element analysis using commercial COMSOL Multiphysics software (COMSOL Inc., Sweden). A 3D steady-state solid mechanics mode was applied to simulate the stress distribution on the scaffolds. The β-TCP ceramic was modeled as a linear elastic material (modulus of elasticity: 110 GPa; Poisson’s ratio: 0.27; density: 3.08 g cm^−3^). The bottom plane was fully fixed when a downward compressive strain of 0.5% was applied to the top plane. After the models were meshed and solved, the stress distribution of the scaffold models could be shown. The computational permeabilities of scaffold models can be obtained by conducting CFD simulations. The 3D peristaltic flow mode was used for CFD simulations. No-slip wall boundary conditions were considered for the scaffold models. The density and viscosity of the flowing medium were assumed to be 1000 kg/m^3^ and 0.001 Pa·s, respectively. The constant flow rate in the inlet was set to 1 mm/min, and the outlet pressure was considered to be zero. The permeability was calculated by the ultimate pressure drop through the scaffold according to Darcy’s law [3]. Due to the anisotropy of the flat-bone-mimetic scaffolds, both mechanical tests and FEA were conducted in the Z and X/Y directions.

**In vitro cell response assessments**

**Cell culture and seeding**

Rat bone marrow mesenchymal stromal cells (rBMSCs; Cyagen, USA) were used to evaluate the in vitro osteogenic differentiation of the scaffolds. Human umbilical vein endothelial cells (HUVECs; ScienCell, USA) were used to assess the angiogenic potential of the scaffolds. The cytocompatibility of bioceramic scaffolds was assessed by the adhesion, viability and proliferation of rBMSCs and HUVECs. The rBMSCs were cultured with Dulbecco’s modified Eagle’s medium (DMEM; Thermo Fisher, USA) containing 10 vol.% fetal bovine serum (FBS; ScienCell, USA). To evaluate the osteogenic differentiation of rBMSCs on the scaffolds, osteoinductive substances (10 mM sodium β-glycerophosphate, 10 nM dexamethasone, and 50 mM vitamin C) were was added to the culture medium. HUVECs were cultivated in endothelial basal medium (ECM; ScienCell, USA) supplemented with 1% FBS and several cytokines (ECGS; ScienCell, USA). After autoclave sterilization, the scaffold samples (6.5 mm × 6.5 mm × 3 mm) were placed into 48-well plates, and 0.5 mL of cell suspension was added to the scaffolds. For the rBMSC cytocompatibility test, 4 × 10^4^ rBMSCs were seeded per scaffold, and 2 × 10^4^ HUVECs were seeded per scaffold. For evaluations of osteogenic differentiation and angiogenic activity, 5 × 10^4^ rBMSCs and HUVECs were seeded per scaffold. The cell-seeded scaffold samples were incubated in a humidified incubator (5% CO_2_, 37 °C). The culture medium was replaced by new medium every other day. The biochemical kits and test instruments were used in accordance with the manufacturers’ guidelines.

**Cytocompatibility evaluation**

After being cultured for the scheduled time, the cell viability was assessed using a Live/Dead kit (Beyotime, China), and fluorescent photographs of live and dead cells were taken with a laser confocal microscope (SP5, Leica, Germany). The cytoskeletons and nuclei of cells on the scaffolds were labeled with a Cell Navigator™ F-Actin Labeling kit (AAT Bioquest, USA) and a DAPI staining kit (Beyotime, China), respectively. The morphology of rBMSCs adhered to the scaffolds was observed by laser confocal microscopy. Cell proliferation was tested using a CCK-8 kit (Beyotime, China).

Alkaline phosphatase **(ALP) activity and calcium nodule formation**

After 7 and 14 days of cell culture, quantitative ALP activity of rBMSCs cultivated on the scaffolds was examined by p-nitrophenyl phosphate assay using an Alkaline Phosphatase Assay Kit (Beyotime, China). The enzymatic activity was determined by detecting the absorbance at 405 nm through an ELISA reader (Vaeioskan Flash, Thermo Fisher, USA). The total proteins were measured with a BCA Protein Assay Kit (Beyotime, China). The total proteins were determined by the absorbance at 562 nm. The ALP activity was denoted as enzymatic activity units per milligram of total protein. Qualitative evaluation of ALP activity of rBMSCs on the scaffolds was examined by staining with a BCIP/NBT kit (Beyotime, China). After culturing for 14 days, the mineralization of rBMSCs was evaluated by staining calcium nodules with an Alizarin Red S staining kit (Beyotime, China). Images of ALP- and ARS-stained scaffold samples were taken by stereomicroscopy.

**Tubule formation**

The tubule formation of HUVECs was assayed with Matrigel matrix (Corning, USA). Briefly, after the HUVECs were cultured on the scaffolds for 3 days, they were resuspended and seeded on the surface of the Matrigel matrix. After incubation for 3 and 6 hours, the cells were labeled with Calcein-AM (Beyotime, China), and tubule formation was observed with an inverted fluorescence microscope (Axioskop 40, Zeiss, Germany).

**Real-time polymerase chain reaction (RT‒PCR)**

RT‒PCR was conducted to analyze the osteogenic gene expression of rBMSCs and angiogenic gene expression of HUVECs. The osteogenic genes included alkaline phosphatase (ALP), collagen type I (Col I), osteocalcin (OCN), osteopontin (OPN) and runt‐related transcription factor 2 (Runx2). The angiogenic genes were platelet endothelial cell adhesion molecule-1 (CD31), endothelial nitric oxide synthase (eNOS), vascular endothelial growth factor receptor 2 (KDR) and vascular endothelial growth factor (VEGF). GAPDH was used as the housekeeping gene. The primer sequences for target genes are presented in Tables S2 and S3. In brief, total RNA was extracted with a HiPure Total RNA micro kit (Magen, China). Subsequently, the RNA was reverse transcribed into complementary DNA (cDNA) utilizing an iScript cDNA synthesis kit (Roche, Germany). Target gene expression was detected using an Applied Biosystems QuantStudio 6 Flex (Life Technologies, USA). The relative amounts of target genes were determined by employing the ΔCt method (ΔCt = Ct (target gene) – Ct (GAPDH)). The relative gene expression levels were determined by calculating 2^−ΔΔCt^.

**Immunofluorescence staining**

Immunofluorescence staining of osteogenic proteins (Ruxn2 and OPN) and angiogenic proteins (CD31) was carried out. After culturing for predetermined times, the cells were immobilized with Paraformaldehyde Fix Solution (Beyotime, China) and permeabilized with Triton X-100 solution (Sigma‒Aldrich, USA). The cells were treated with a solution of primary antibodies (Affinity, USA) overnight at 4 ℃, and subsequently incubated with the secondary antibody (Affinity, USA) and DAPI for 15 min at room temperature. The cells were observed under a laser confocal microscope. Semiquantitative evaluations of protein expression were performed with Image-Pro Plus 6.0 software (Media Cybernetic, USA).

**In vivo experiment**

The critical-sized cranial defect model of rabbits was developed to assess the bone regenerative capacity of flat-bone-mimetic bioceramic scaffolds. The experiments on the rabbits were approved by the Laboratory Animal Ethics Committee of Guangzhou Huateng Biomedical Technology (approval number: HTSW220418). New Zealand white rabbits (male, 2-2.5 kg) were randomly divided into three groups: Cross-hatch, Gyr-Comp and Gyr-Tub (n = 4 per group at each time point). The rabbits were anesthetized with pentobarbital sodium, and a skin incision was created to expose the cranium. A dental drill was used to create two 10-mm diameter defects on the exposed cranium (**Fig. S12**). The scaffolds (Ø10 mm × 3 mm) were implanted into the defects, and the incision was sutured layer upon layer. After 6 and 12 weeks, the rabbits were sacrificed with CO_2_ suffocation, and the scaffold implants together with the cranium were harvested. μ-CT (μCT-100; SCANCO Medical AG, Switzerland) was used to analyze bone regeneration inside the scaffold implants. The voltage, current, resolution and exposure time were 70 kV, 30 μA, 30 μm and 300 ms, respectively. Visualization and quantitative analysis of the bone generation fraction (bone volume over total volume, abbreviated as BV/TV) were conducted by Avizo software (Thermo Fisher, USA). The bone mineral density was analyzed with Evaluation V6.5-3 software (SCANCO Medical AG, Switzerland). The samples were decalcified with 10% ethylenediaminetetraacetic acid (EDTA) solution, embedded in paraffin and sectioned to obtain sections with a thickness of 5 μm. Histological analyses, including hematoxylin-eosin (HE) staining, Masson’s staining, and immunohistochemical staining of CD31 and von Willebrand factor (VWF), were performed to observe the formation of new bone tissues and blood vessels in the defect sites.

**Statistical analysis**

All tests were performed independently at least four times, and the data are presented as the mean ± standard deviation. Student’s t test was used to analyze the difference between experimental groups, and a statistically significant difference was considered when * p < 0.05.

**Supplementary figures and tables**


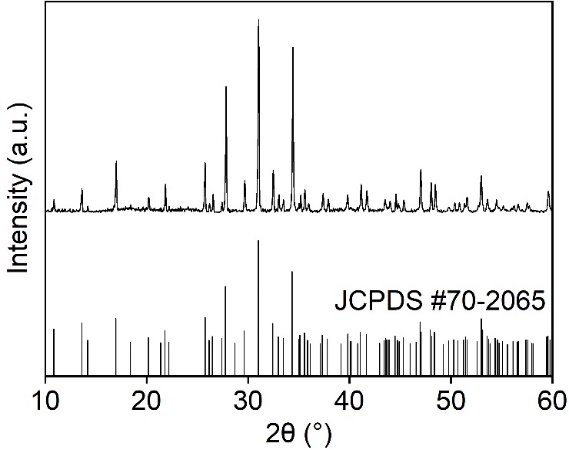


**Fig. S1.** XRD pattern of bioceramic scaffolds.


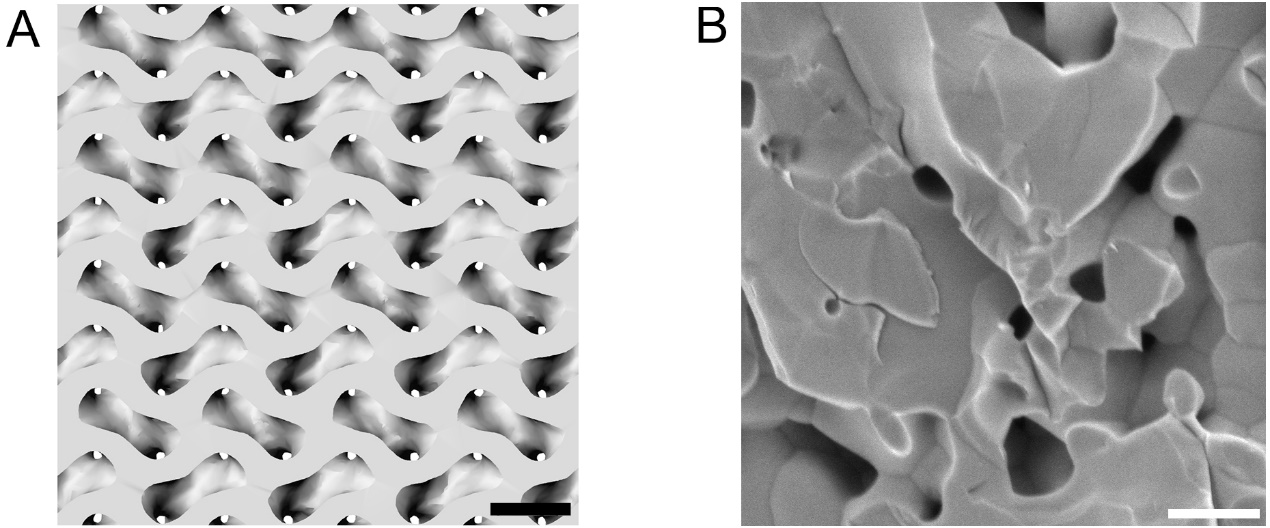


**Fig. S2**. (A) μ-CT photograph of the cross section of the gyroid porous layer of Gyr-Tub scaffolds. Scale bar = 1 mm. (B) SEM image of the fractured surface of the bioceramic scaffolds. Scale bar = 2 μm.


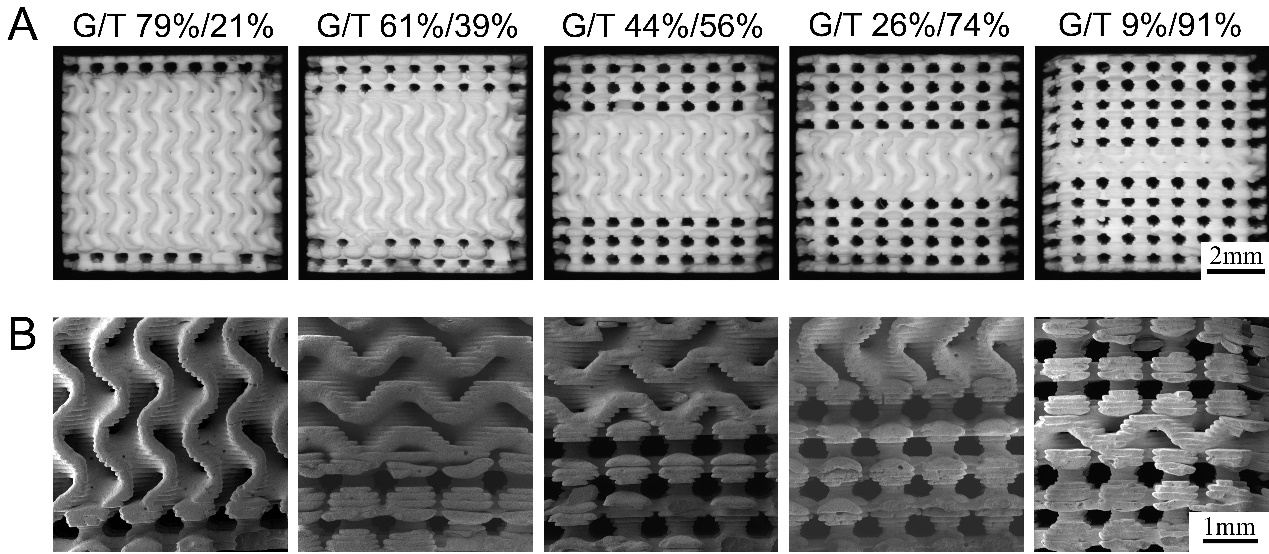


**Fig. S3**. Stereomicrographs (A) and SEM photographs (B) of Gyr-Tub scaffolds with different thickness ratios of gyroid porous layer to tubular porous layer (G/T).


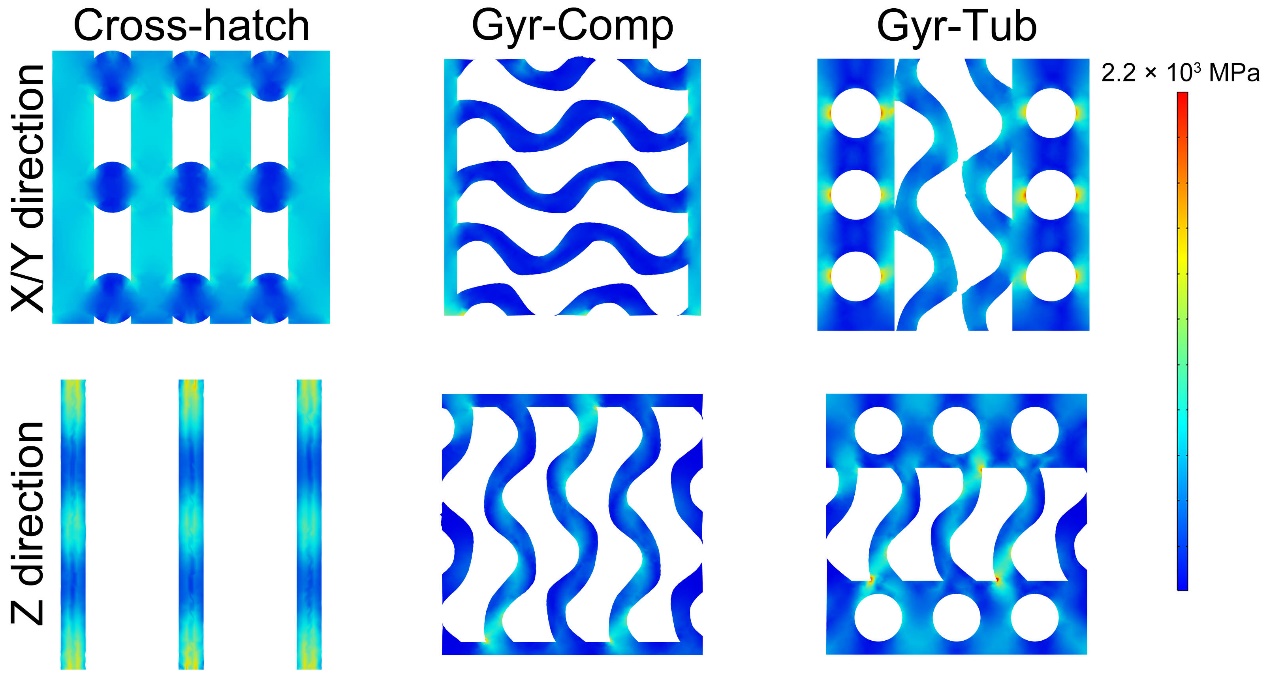


**Fig. S4**. Von Mises stress distributions of the section of the Cross-hatch, Gyr-Comp and Gyr-Tub scaffold models.


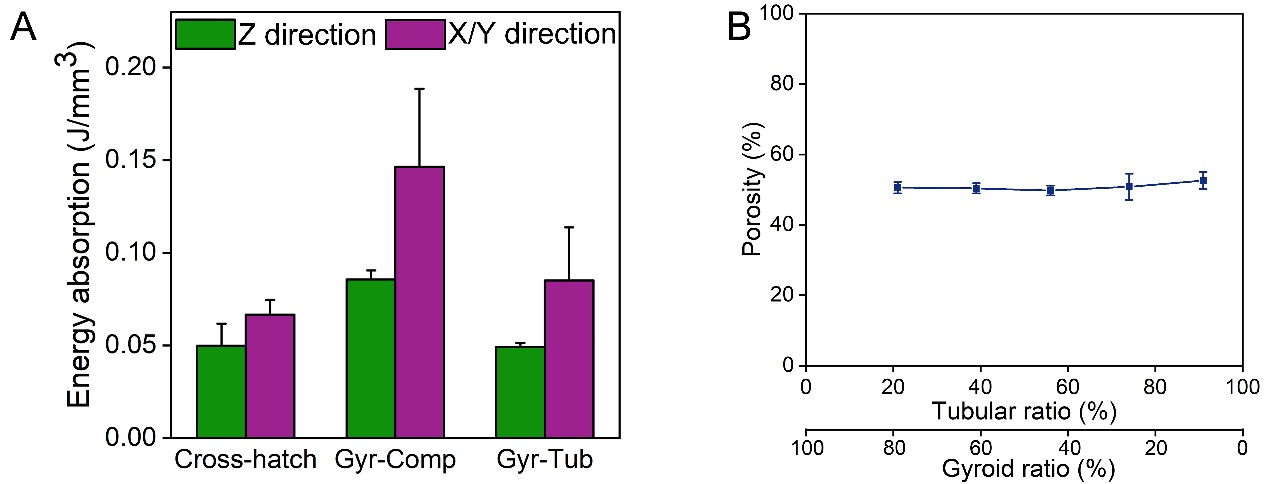


**Fig. S5**. (A) Energy absorption capacity of scaffolds subjected to compression. (B) Apparent porosity of Gyr-Tub bioceramic scaffolds as a function of thickness ratio of tubular porous layer or gyroid porous layer.


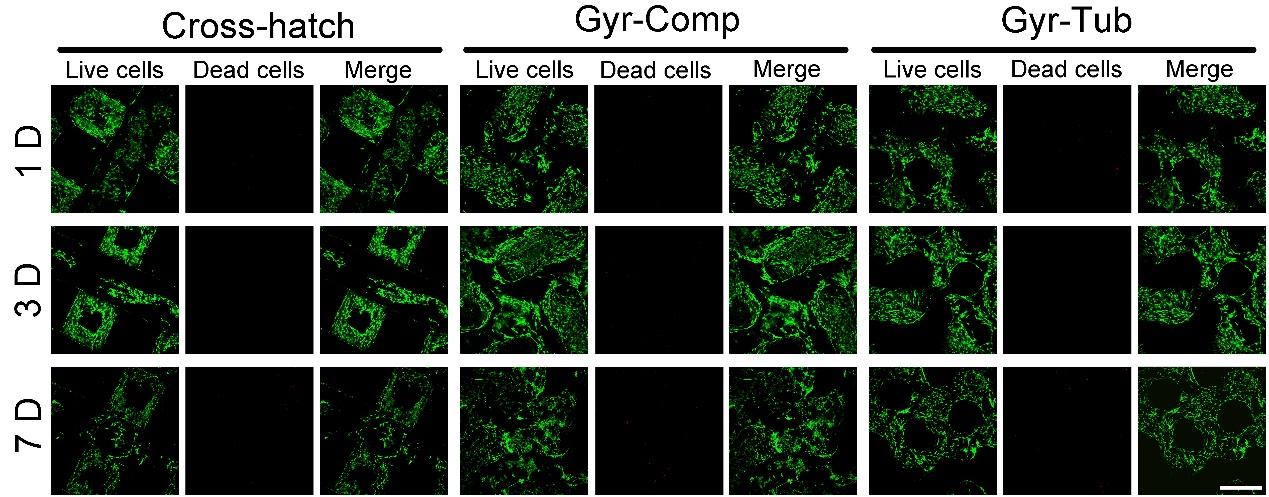


**Fig. S6**. Fluorescent photographs of live cells and dead cells on the scaffolds after culturing for 1, 3 and 7 days. Scale bar = 500 μm.


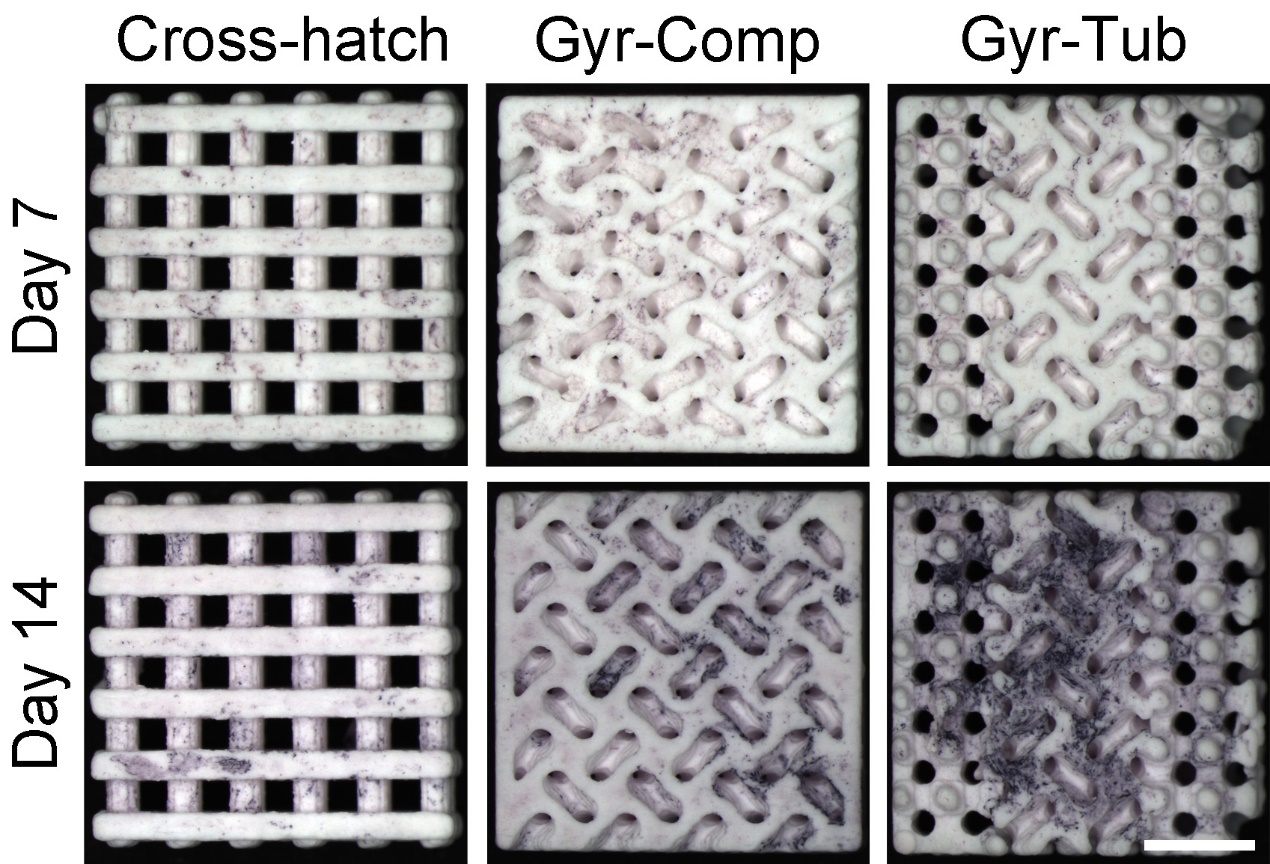


**Fig. S7**. Photographs of ALP staining of rBMSCs cultured on the Cross-hatch, Gyr-Comp and Gyr-Tub scaffolds for 7 and 14 days. Scale bar = 2 mm.


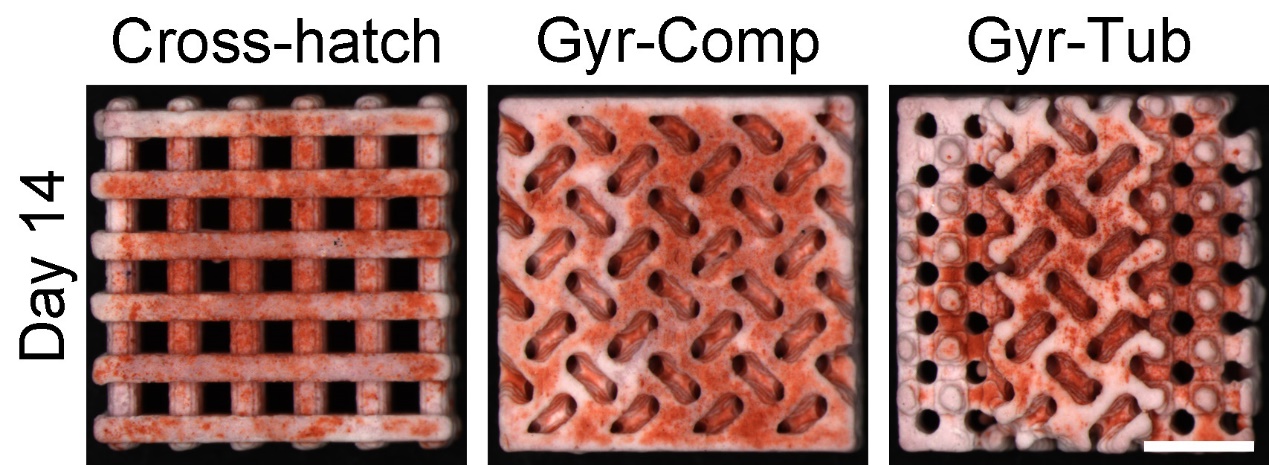


**Fig. S8**. Photographs of Alizarin Red S staining of rBMSCs cultured on the Cross-hatch, Gyr-Comp and Gyr-Tub scaffolds for 14 days. Scale bar = 2 mm.


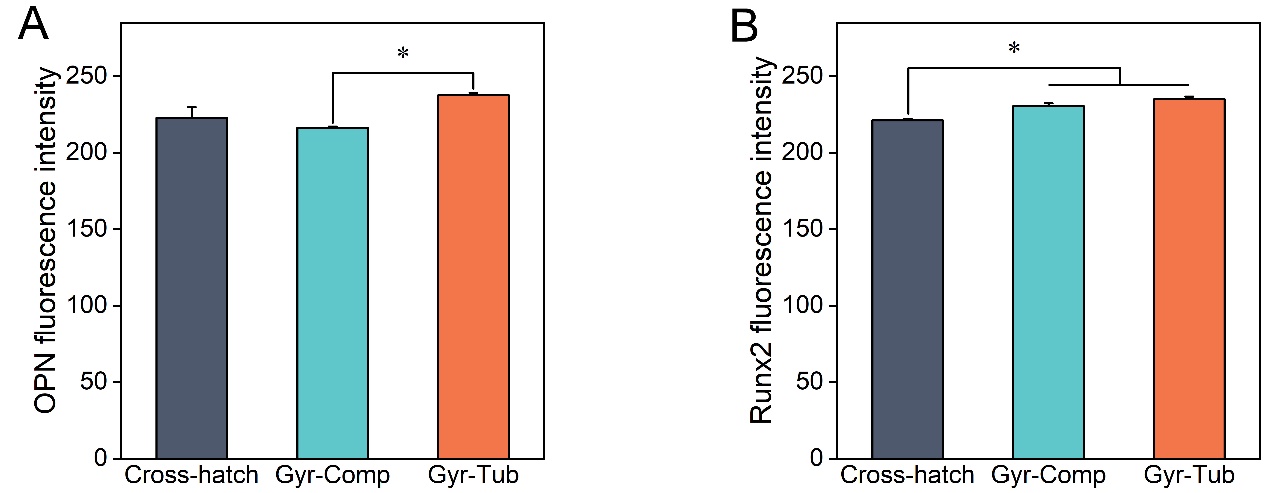


**Fig. S9**. Fluorescence intensity of OPN and RUNX2 of rBMSCs cultured on the scaffolds for 7 days.


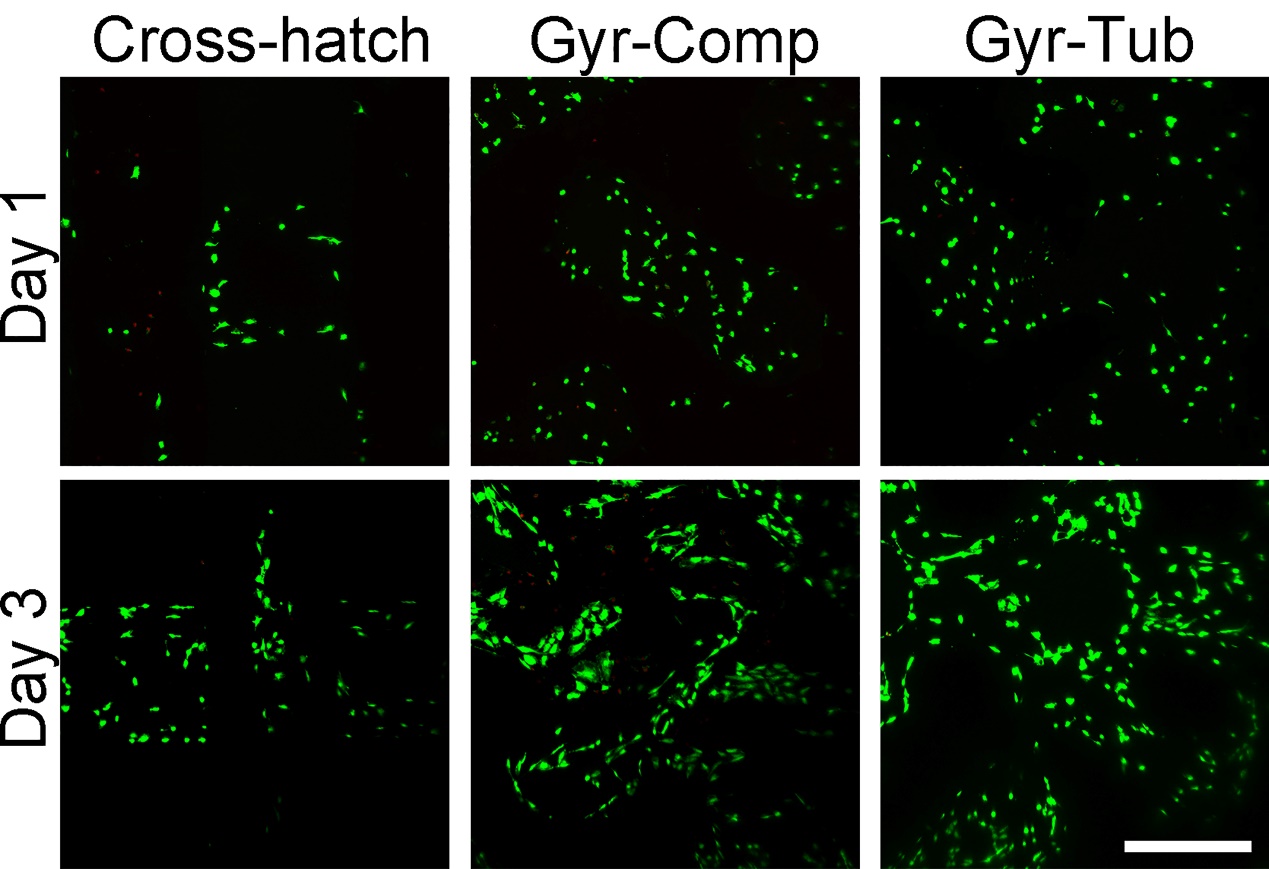


**Fig. S10**. Fluorescent photographs of Live/dead staining of HUVECs cultured on the scaffolds for 1 and 3 days. Scale bar = 500 μm.


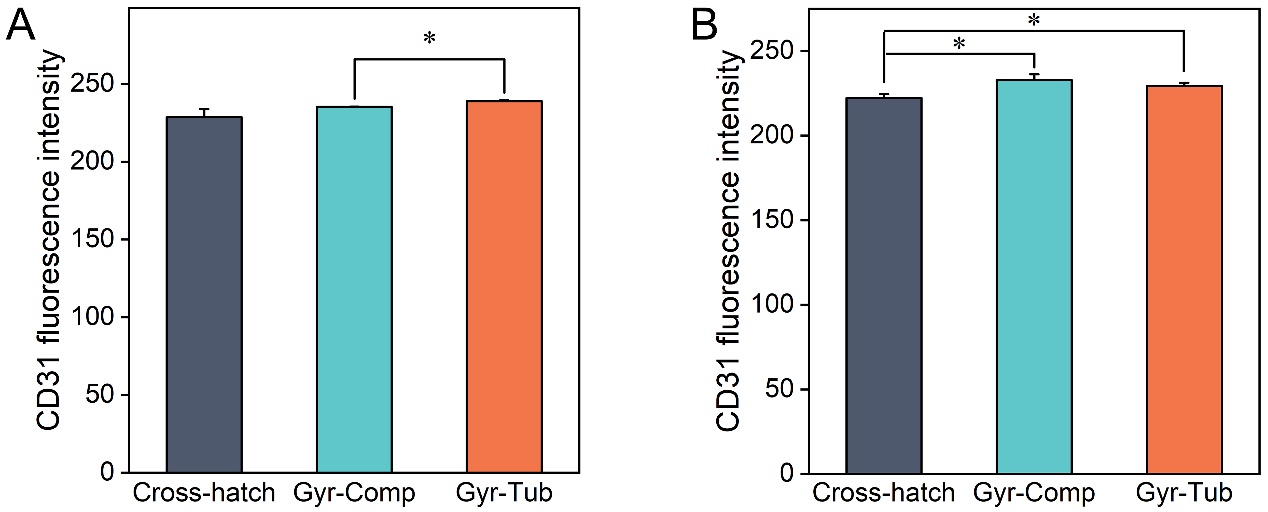


**Fig. S11**. Fluorescence intensity of CD31 of HUVECs cultured on the scaffolds for 3 days (A) and 5 days (B).


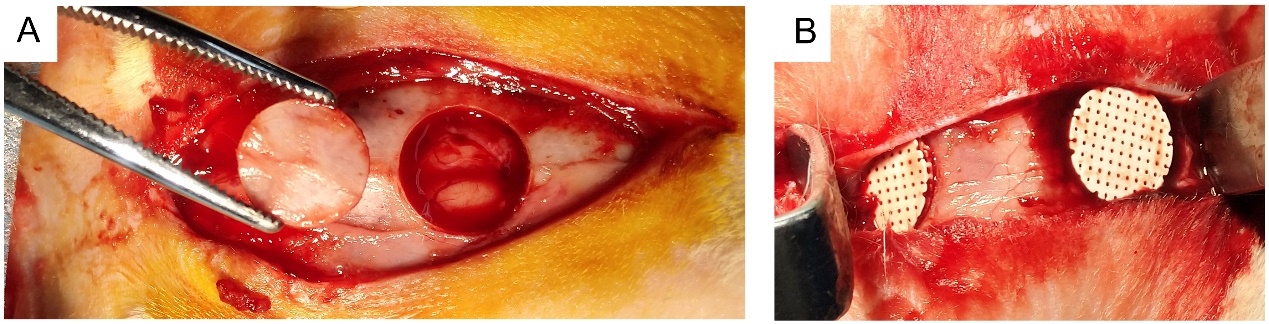


**Fig. S12**. (A) Creation of rabbit cranial defect. (B) Implantation of scaffold samples into the cranial defects.


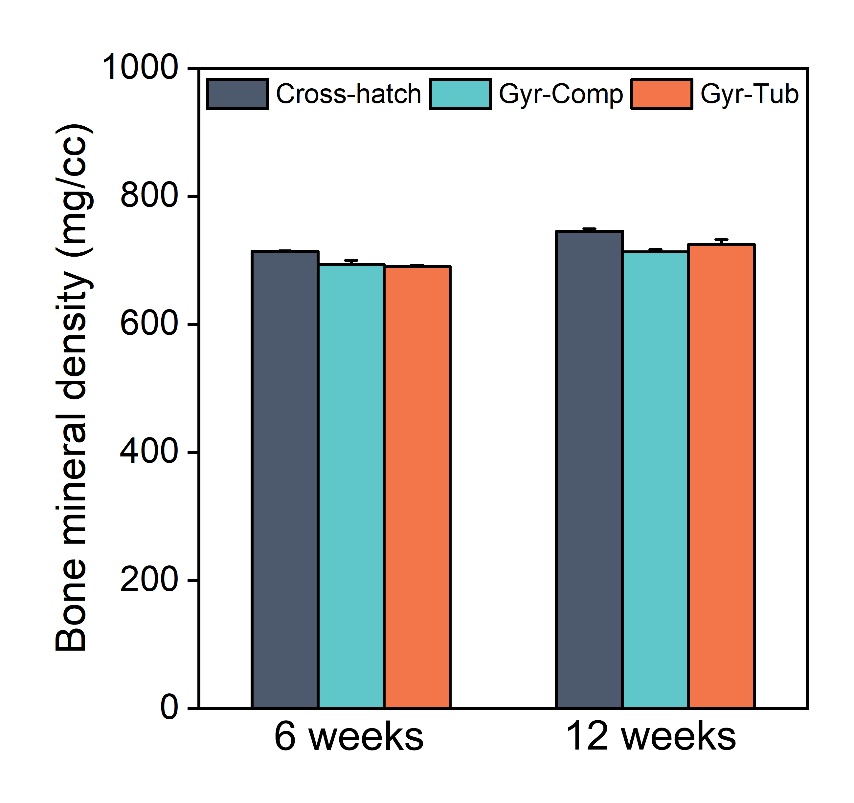


**Fig. S13**. Bone mineral density of bone defects after implantation of bioceramic scaffolds for 6 and 12 weeks.


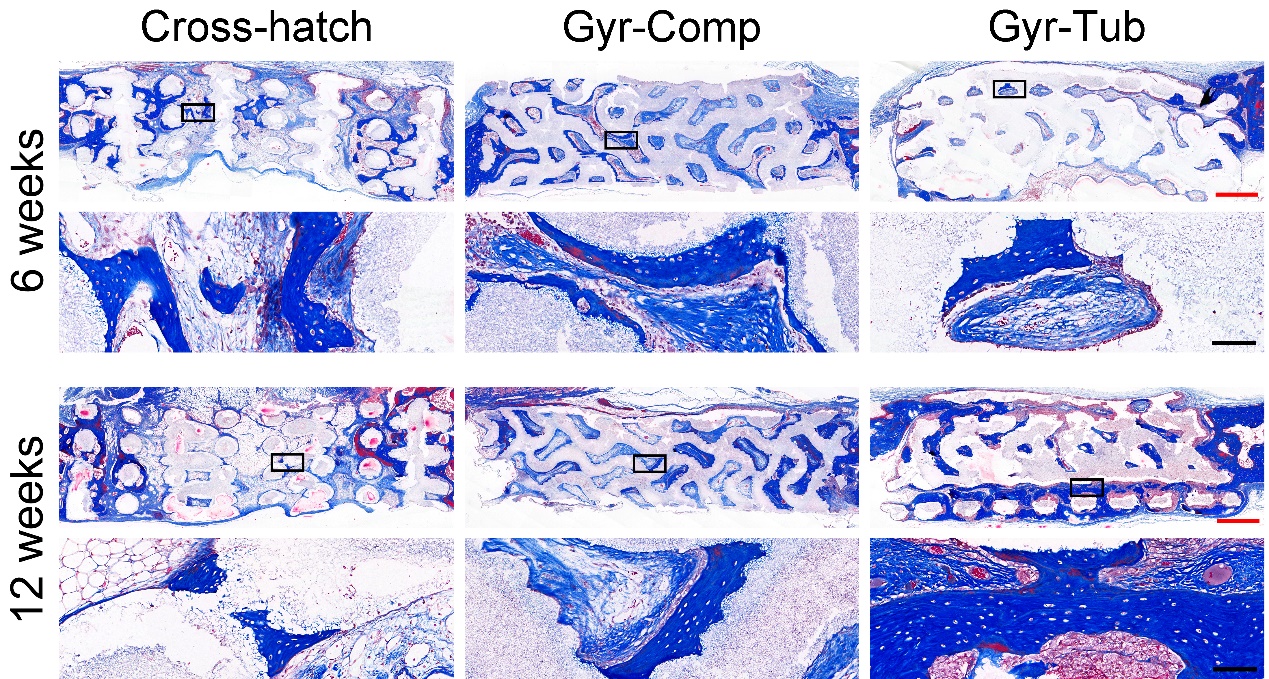


**Fig. S14**. Histological examination by Masson’s staining after implantation for 6 and 12 weeks. Red scale bar = 1 mm, black scale bar = 100 μm.


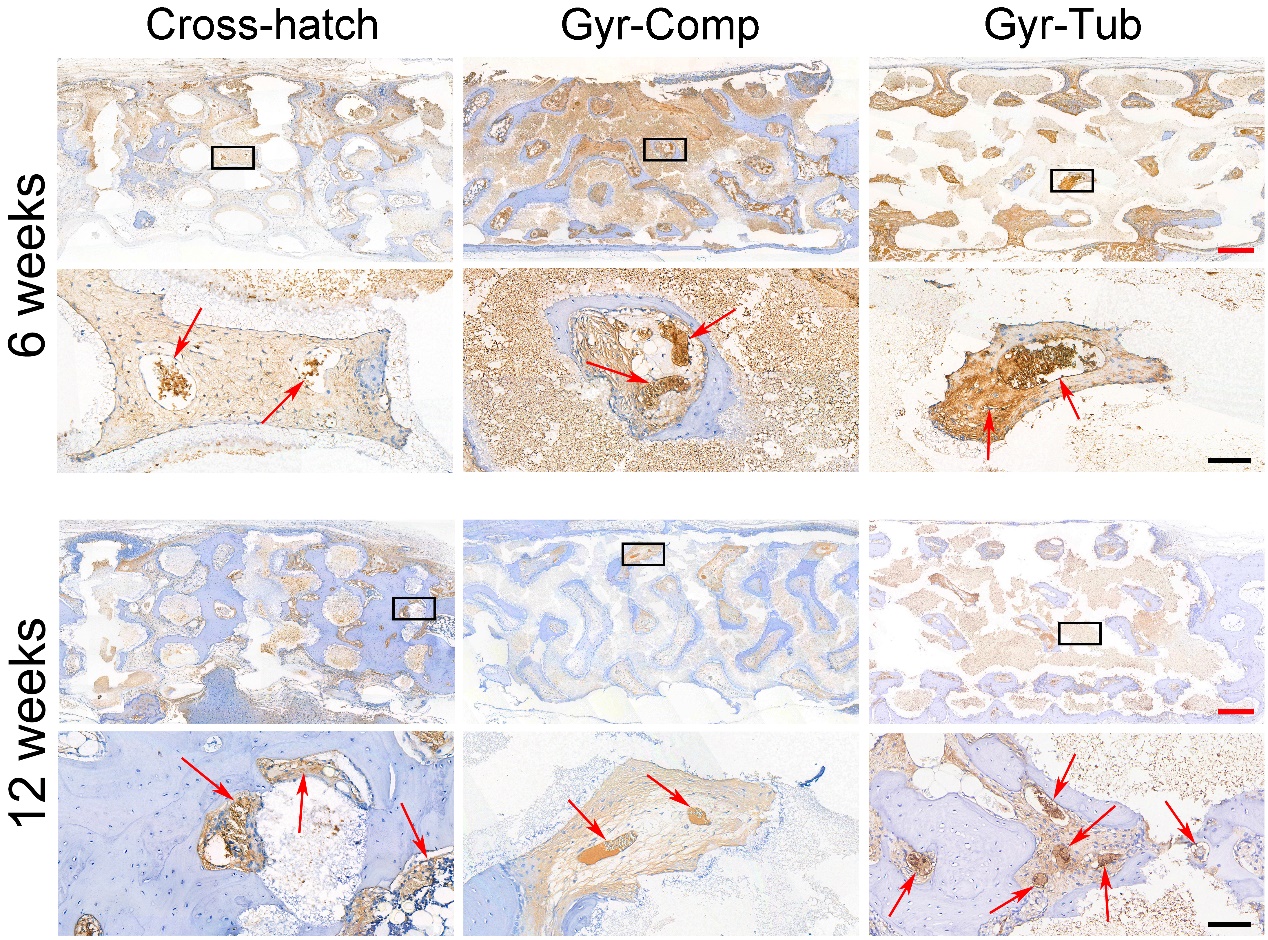


**Fig. S15**. CD31 immunohistochemical images after implantation for 6 and 12 weeks. Red arrows point to the CD31-positive blood vessels. Red scale bar = 500 μm, black scale bar = 200 μm.


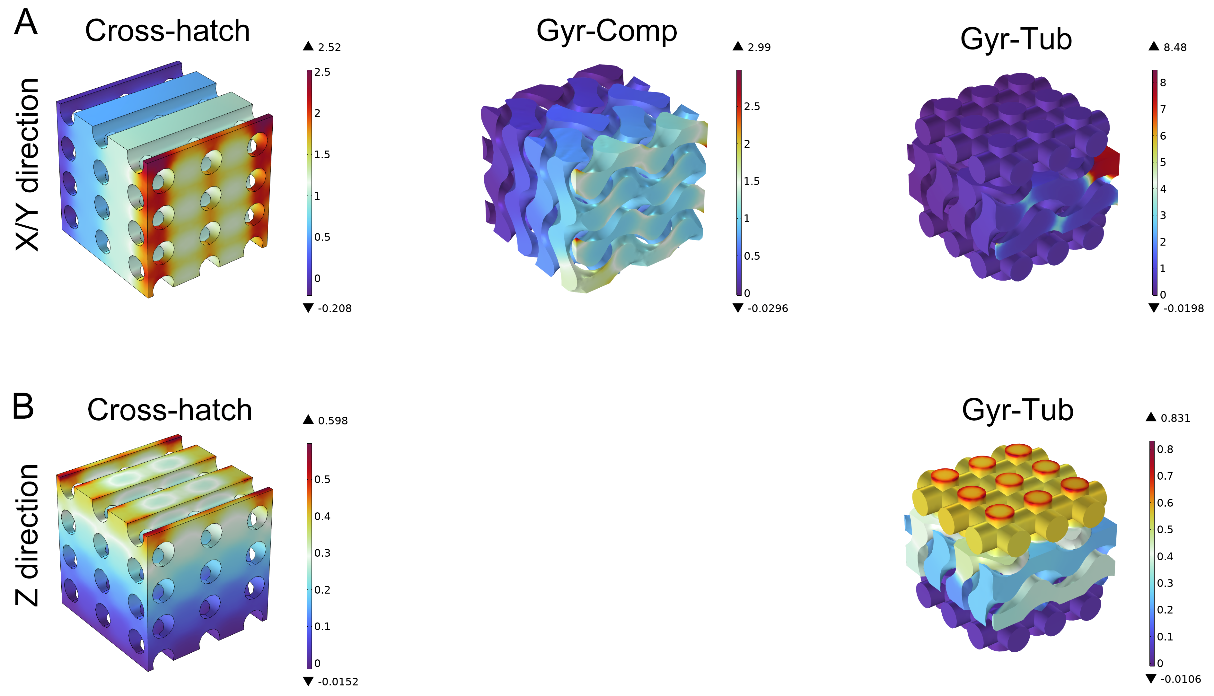


**Fig. S16**. CFD simulation of the pressure distributions of Cross-hatch, Gyr-Comp and Gyr-Tub scaffolds in different flow directions (A: X/Y direction; B: Z direction).

**Table S1**. Permeabilities of Cross-hatch, Gyr-Comp and Gyr-Tub scaffolds in different directions, which were obtained from the CFD simulation.

| Scaffolds | X/Y-direction permeability (m^2^) | Z-direction permeability (m^2^) |
| --- | --- | --- |
| Cross-hatch | 1.30 × 10^−9^ | 4.17 × 10^−9^ |
| Gyr-Comp | 1.27 × 10^−9^ | N.D. |
| Gyr-Tub | 1.02 × 10^−9^ | 2.14 × 10^−9^ |


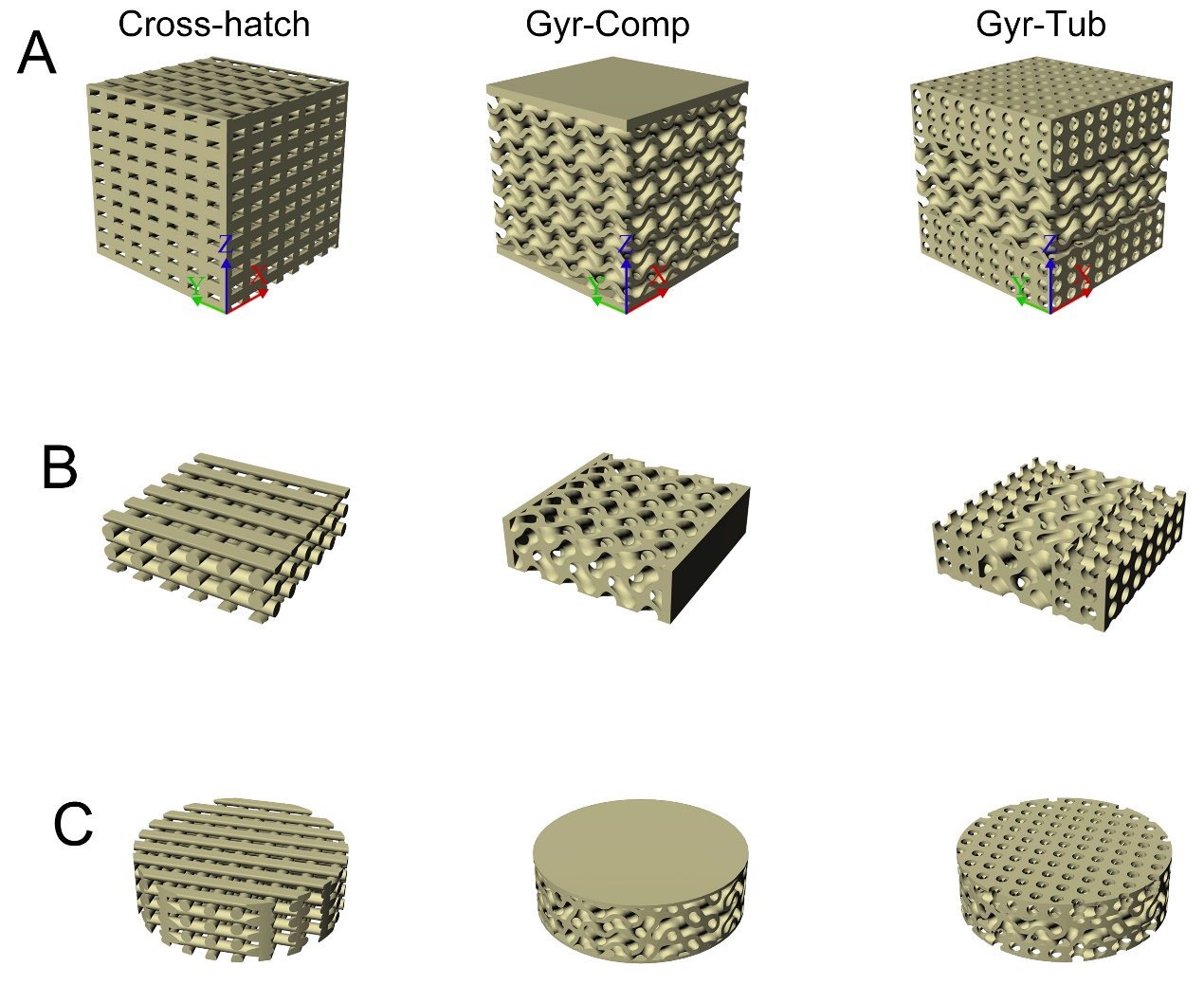


**Fig. S17**. (A) Scaffold samples (8 mm × 8 mm × 8 mm) used for mechanical tests; the X direction was identical to the Y direction. The mechanical tests were performed along the Z direction and X/Y direction. (B) Scaffold samples (6.5 mm × 6.5 mm × 3 mm) used for cell response assessments. (C) Scaffold samples (Φ 10 mm × 3 mm) used for in vivo implantation.

**Table S2**. Primer sequences of osteogenic genes used for RT‒PCR in the study.

| Gene | Primer sequences | |
| --- | --- | --- |
| GAPDH | 5’-3’ | GTCCATGCCATCACTGCCACTC |
|  | 3’-5’ | CGCCTGCTTCACCACCTTCTTG |
| ALP | 5’-3’ | CACTTCAGGGTCAGGTTTCCACAG |
|  | 3’-5’ | GAGGTTGAGGTTACAGTCAGCACAC |
| Col I | 5’-3’ | TCACCAGACGCAGAAGTCATAGGAG |
|  | 3’-5’ | GGAGACCACGAGGACCAGAAGG |
| Runx2 | 5’-3’ | GCACTATCCAGCCACCTTCACTTAC |
|  | 3’-5’ | TCCATCAGCGTCAACACCATCATTC |
| OCN | 5’-3’ | GTAGTGACGGAGAAGCAGCAGATG |
|  | 3’-5’ | TTCCAGACGGTCCAGGCAGATG |
| OPN | 5’-3’ | GCCGAGGTGATAGTGTGGTTTATGG |
|  | 3’-5’ | ATAATCTGGACTGCTTGTGGCTGTG |

**Table S3**. Primer sequences of osteogenic genes used for RT‒PCR in the study.

| Gene | Primer sequences | |
| --- | --- | --- |
| GAPDH | 5’-3’ | CCACATCGCTCAGACACCAT |
|  | 3’-5’ | GGCAACAATATCCACTTTACCAG |
| VEGF | 5’-3’ | TGCGGATCAAACCTCACCA |
|  | 3’-5’ | CAGGGATTTTTCTTGTCTTGCT |
| eNOS | 5’-3’ | TGATGGCGAAGCGAGTGAAG |
|  | 3’-5’ | ACTCATCCATACACAGGACCC |
| KDR | 5’-3’ | GTGATCGGAAATGACACTGGAG |
|  | 3’-5’ | CATGTTGGTCACTAACAGAAGCA |
| CD31 | 5’-3’ | ACGCTGGTGCTCTATGCAAG |
|  | 3’-5’ | TCAGTTGCTGCCCATTCATCA |

**References**

1. He F, Qian G, Ren W, Ke J, Fan P, Shi X, Cheng Y, Wu S, Deng X, Ye J. Preparation and characterization of iron/β-tricalcium phosphate bio-cermets for load-bearing bone substitutes. *Ceram Int*. 2017;43(11):8348–8355.

2. Zhang Y, Zhang Q, He F, Zuo F, Shi X. Fabrication of cancellous-bone-mimicking β-tricalcium phosphate bioceramic scaffolds with tunable architecture and mechanical strength by stereolithography 3D printing. *J Eur Ceram Soc*. 2022;42(14):6713–6720.

3. Truscello S, Kerckhofs G, Van Bael S, Pyka G, Schrooten J,Van Oosterwyck H. Prediction of permeability of regular scaffolds for skeletal tissue engineering: a combined computational and experimental study. *Acta Biomater*. 2012;8(4):1648–1658.
